# SNX27 suppresses SARS-CoV-2 infection by inhibiting viral lysosome/late endosome entry

**DOI:** 10.1073/pnas.2117576119

**Published:** 2022-01-13

**Authors:** Bo Yang, Yuanyuan Jia, Yumin Meng, Ying Xue, Kefang Liu, Yan Li, Shichao Liu, Xiaoxiong Li, Kaige Cui, Lina Shang, Tianyou Cheng, Zhichao Zhang, Yingxiang Hou, Xiaozhu Yang, Hong Yan, Liqiang Duan, Zhou Tong, Changxin Wu, Zhida Liu, Shan Gao, Shu Zhuo, Weijin Huang, George Fu Gao, Jianxun Qi, Guijun Shang

**Affiliations:** ^a^Shanxi Academy of Advanced Research and Innovation, Taiyuan 030032, China;; ^b^Shanxi Provincial Key Laboratory for Major Infectious Disease Response, Taiyuan 030012, China;; ^c^Chinese Academy of Sciences Key Laboratory of Pathogen Microbiology and Immunology, Institute of Microbiology, Chinese Academy of Sciences, Beijing 100101, China;; ^d^Institutes of Biomedical Sciences, Shanxi University, Taiyuan 030006, China;; ^e^Signet Therapeutics Inc, Shenzhen 518000, China;; ^f^Division of HIV/AIDS and Sex-Transmitted Virus Vaccines, National Institutes for Food and Drug Control, Beijing 102629, China

**Keywords:** ACE2, SNX27, retromer, RBD, SARS-CoV-2

## Abstract

After binding to its cell surface receptor angiotensin converting enzyme 2 (ACE2), severe acute respiratory syndrome coronavirus 2 (SARS-CoV-2) enters the host cell through directly fusing with plasma membrane (cell surface pathway) or undergoing endocytosis traveling to lysosome/late endosome for membrane fusion (endocytic pathway). However, the endocytic entry regulation by host cell remains elusive. Recent studies show ACE2 possesses a type I PDZ binding motif (PBM) through which it could interact with a PDZ domain-containing protein such as sorting nexin 27 (SNX27). In this study, we determined the ACE2-PBM/SNX27-PDZ complex structure, and, through a series of functional analyses, we found SNX27 plays an important role in regulating the homeostasis of ACE2 receptor. More importantly, we demonstrated SNX27, together with retromer complex (the core component of the endosomal protein sorting machinery), prevents ACE2/virus complex from entering lysosome/late endosome, resulting in decreased viral entry in cells where the endocytic pathway dominates. The ACE2/virus retrieval mediated by SNX27–retromer could be considered as a countermeasure against invasion of ACE2 receptor-using SARS coronaviruses.

## Significance

We here established the interaction between PDZ binding motif (PBM) at the C terminal of ACE2 and PDZ domain of sorting nexin 27 (SNX27) and solved the crystal structure of ACE2-PBM/SNX27-PDZ complex. Together with retromer complex, SNX27 was found to regulate the homeostasis of cell surface ACE2 under physiological conditions. When endocytic pathway was used during SARS-CoV-2 infection, SNX27-retromer sorts ACE2/SARS-CoV-2 complex at early endosome and prevents it from entering lysosome/late endosome, inhibiting the cell entry of the virus. These findings add substantially to the current understanding of the important role of cytosolic tail of ACE2 during the invasion of SARS-CoV-2, and it could be used as a new therapeutic target for drug development.

Severe acute respiratory syndrome coronavirus 2 (SARS-CoV-2) causing the disease COVID-19 has evolved rapidly and posed a grave threat for the public health (1–7). It is thus urgent to unravel the viral infection mechanism to expedite the process of drug discovery, vaccine, and new therapy developments. SARS-CoV-2 recognizes host receptor ACE2 by using the receptor binding domain (RBD) of its spike protein (S protein) (8, 9), which is pretty similar to SARS-CoV that emerged in 2002 (10). After attaching to the cell surface and binding to the ACE2 receptor, SARS-CoV-2 virus uses two different entry pathways to get into cytosol and release its genetic materials leading to successful infection (11). One route, also called the cell surface pathway (or early pathway) (12), is that virus directly fuses with plasma membrane with the help of cell surface proteases such as type II transmembrane serine protease, TMPRSS2, which processes the S protein to initiate membrane fusion (11). The other one is through virus-triggered endocytic pathway (late pathway), which is involved in the trafficking of virus to the lysosome/late endosome in which the proteases such as cathepsin L (CTSL) prime S protein for membrane fusion (11, 13). The supposed two-pathway entry strategy adopted by SARS-CoV-2 enables it to be much more transmissible than SARS-CoV (14).

When cell surface receptors are stimulated by their ligands or virus, they undergo endocytosis, and the internalized receptors are then transported to early endosomes (EEs) (15). The fates of the endosomal receptors are determined by sorting to different cellular compartments. Parts of internalized receptors are retrieved to recycle endosomes by which they will travel back to plasma membrane, while others are delivered to late endosomes fused with lysosome for degradation (16). SNX27, one of the sorting nexin (SNX) family members, regulates the trafficking of endosomal receptors (protein cargoes) from EEs to recycling endosomes (17–19). SNX27 contains three domains: the PDZ (PSD-95, Dlg, and ZO-1) domain at N terminus, the PX (phox homology) domain in the middle, and the FERM (4.1, ezrin, radixin, moesin) domain at C terminus. Both the FERM domain and the PX domain could associate with membrane (20, 21), while the PDZ domain and the FERM domain recognize PDZ binding motif (PBM)-containing cargoes and NPXY or NXXY motif-containing cargoes, respectively. The retromer is an evolutionary conserved protein complex which is vital for directing cargoes to recycling endosomes or trans-Golgi network retrograde pathway (22). SNX27 could engage with retromer complex and sort cargoes to the tubular structure decorated by recycling machinery (22, 23). The retromer complex consists of its core components (VPS35, VPS29, and VPS26) and other associated subcomplexes (24). Together with SNX27, retromer complex plays an essential role in regulating the cell surface receptor homeostasis, disrupting of which could result in receptor lysosome/late endosome degradation and neurodegenerative disease such as Alzheimer’s Disease (25–27). The SARS-CoV or SARS-CoV-2 coronavirus receptor ACE2 belongs to type I transmembrane protein, and it owns a large extracellular region at the N terminus and one short intracellular C-terminal tail. The extracellular region of ACE2 consists of a metallopeptidase domain directly engaging spike protein of coronaviruses and a collectrin domain functioning as a dimerization domain (28, 29). In contrast with extracellular region, the function of intracellular tail of ACE2 is poorly understood, especially for the role it plays during the trafficking of internalized ACE2 receptor. Recent bioinformatic analysis shows the tail of ACE2 contains a PBM (30), and binding assay shows the PBM could bind to several PDZ-containing proteins such as Na^+^/H+ exchanger regulatory factor (NHERF) family, SHANK1, and SNX27 (31, 32). Meanwhile, several studies using high-throughput CRISPR screening reported that SNX27 and retromer complex play important roles in regulating SARS-CoV-2 entry, while the detailed mechanism remains elusive (33–36). Here, we present the complex structure of ACE2-PBM/SNX27-PDZ and find that SNX27 could regulate the abundance of ACE2 on the cell surface, on one hand. On the other hand, SNX27 directs the trafficking of ACE2/virus, voiding its lysosome/late endosome entry in Huh7 cells. These data highlight SNX27 as a novel regulator for ACE2 trafficking and viral infection and provide the possibility of utilization of SNX27-retromer as an antiviral therapeutic target.

## Results

### Biochemical and Crystallographic Characterization of Interaction between ACE2 and SNX27.

**Fig. 1.**
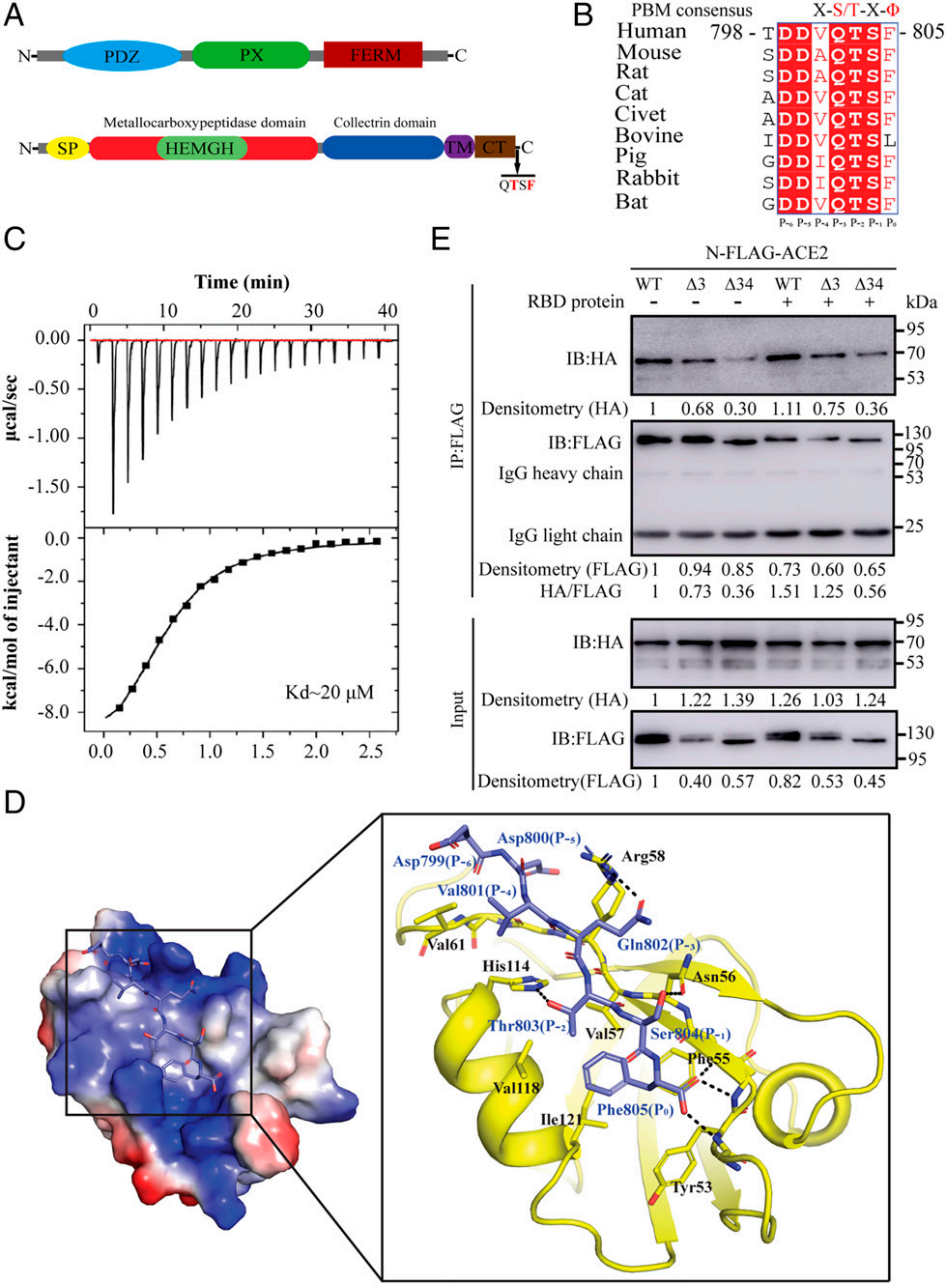
Identification of interaction between ACE2 and SNX27. (*A*) Domain structures of human SNX27 (*Top*) and ACE2 (*Bottom*). (*B*) Sequence alignment and conservation of the C-terminal region of ACE2 from different species. Residue numbers are based on human ACE2. The position of residues in the PBM is shown. (*C*) ITC data for titration of human ACE2-PBM peptide into PDZ domain of human SNX27. (*Top*) Raw data. (*Bottom*) Integrated heat data. (*D*) Crystal structure of ACE2-PBM/SNX27-PDZ complex. (*Left*) Electrostatic representation of the SNX27-PDZ domain bound to ACE2-PBM peptide (sky blue sticks). (*Right*) Detailed interactions of the SNX27-PDZ domain (yellow) bound to ACE2-PBM peptide (sky blue sticks). The residues involved are labeled. H bonds are shown in dashed line. (*E*) Co-IP of HEK293T transiently coexpressing SNX27-HA with FLAG-ACE2, FLAG-ACE2Δ3, or FLAG-ACE2Δ34 with or without RBD protein treatment. The dots were quantified based on the densitometry measured by ImageJ, and the values are shown after normalization to the dot density of wild-type ACE2 or SNX27.

During the preparation of our paper, several studies reported the existence of PBM in the C terminus of ACE2 (30, 32, 36), and it could bind to pairs of PDZ-containing proteins, among which SNX27 is the strongest binder for ACE2/PBM (32, 37). Actually, sequence alignment of ACE2 from mammals shows PBM of ACE2 is well conserved and belongs to type I PBM (S/T-X-Ø, where X denotes any residue, and Ø denotes hydrophobic residue) (Fig. 1 *A* and *B*). In order to further confirm the binding capability of ACE2-PBM to the PDZ domain of SNX27, we synthesized short peptide (-DDVQTSF-COO^−^) based on human ACE2-PBM (residues from 799 to 805) and performed an isothermal titration calorimetry (ITC) experiment to examine the direct binding between ACE2-PBM and the PDZ domain of SNX27. As expected, ITC result shows ACE2-PBM directly engages the PDZ domain of SNX27 with a binding affinity of 20 μM (*K*_D_) (Fig. 1*C*). Although it is a little weaker than the binding affinity measured by two recent experiments (*K*_D_ ≈ 3 and 5 μM, respectively) (32, 37), our ITC result is in the normal range of canonical PBM/PDZ interaction (1 μM to 100 μM) (19, 38–41). Based on previous knowledge, the positions P_3_ and P_5_ in the PBM should be acidic residues forming an acidic clamp interacting with the conserved residue Arg58 in the PDZ domain of SNX27 and especially the acidic residue at position P_-3_ is essential for the binding (38). However, the position P_-3_ in the PBM of ACE2 is glutamine (Gln802), a neutral residue, which raises the question how this substitution makes the binding possible. In order to unravel the molecular mechanism governing the strong binding of ACE2-PBM and SNX27-PDZ, we determined the structure of human ACE2-PBM/SNX27-PDZ complex at 1.29 Å (*SI Appendix*, Fig. S1). The complex structure shows PBM peptide adopts an extended β strand forming pairs of backbone hydrogen bonds with the PDZ domain (Fig. 1*D*), and the overall interaction mode is similar to other PBM/SNX27 complex structures. The highly conserved residue T803 (P_-2_ position) forms a hydrogen bond with residue H114 from the PDZ domain, and the C-terminal carboxyl group forms hydrogen bonds with nitrogens of backbone from SNX27 (GYGF_52–55_ motif), which are the canonical interactions observed in type I PBM/PDZ complex. Hydrophobic residue V801 (P_-4_ position) from PBM packs against V161 from the PDZ domain. The backbones of D799 (P_-6_ position) and D800 (P_-5_ position) form hydrogen bonds with the corresponding backbones from the PDZ domain, whereas their side chains have no apparent interactions with the PDZ domain. Of note is that Gln802 (P_-3_ position) from PBM forms a hydrogen bond with conserved Arg58 from SNX27 instead of the charge–charge interaction found in other SNX27-PBM complex structures. The conserved Ser804 (P_-1_ position) in ACE2-PBM is hydrogen-bonded with Asn56 of SNX27, while, in other PBMs, this residue is usually replaced by hydrophobic residue which apparently lacks hydrogen bonding with the PDZ domain (38). Furthermore, the bulky side chain of Phe805 (P_0_ position) at the very C-terminal end of ACE2-PBM could form a stronger hydrophobic interaction with residues such as V57, F55, V118, and I121 from the PDZ domain. Taken together, these factors contribute to the strong binding between ACE2-PBM and SNX27-PDZ, even in the absence of acidic clamp in the PBM sequence.

Next, we carried out the coimmunoprecipitation (co-IP) assay to further confirm our ITC and crystallographic results. We coexpressed full-length N-terminal Flag-tagged ACE2 and HA-tagged SNX27 in human embryonic kidney cells (HEK293T) and coimmunoprecipitated the complex using Flag beads. The co-IP result clearly shows the interaction between these two proteins (Fig. 1*E*). At the same time, we also made another two constructs with the last three key PDZ binding residues (ACE2Δ3) or the whole cytosolic tail of ACE2(ACE2Δ 34) and conducted the co-IP assay. In contrast to wild-type ACE2, ACE2Δ3 decreased but didn’t abolish its binding with SNX27, whereas ACE2Δ 34 almost abolished the binding, which indicates the extra binding elements may exist in the cytosolic tail besides the PBM. To test whether the binding between ACE2 and SNX27 is operated in a signal-dependent manner, we used RBD protein to treat cells to trigger ACE2 internalization and examined their binding by co-IP assay. In fact, the RBD stimulation not only enhanced the binding between wild-type ACE2 and SNX27, but the binding between ACE2Δ3 and SNX27 also increased (Fig. 1*E*). The signal-dependent binding enhancement suggests the ACE2/SNX27 complex is regulated and formed during ACE2 intracellular trafficking.

### SNX27 Regulates ACE2 Protein Cell Surface Homeostasis.

**Fig. 2.**
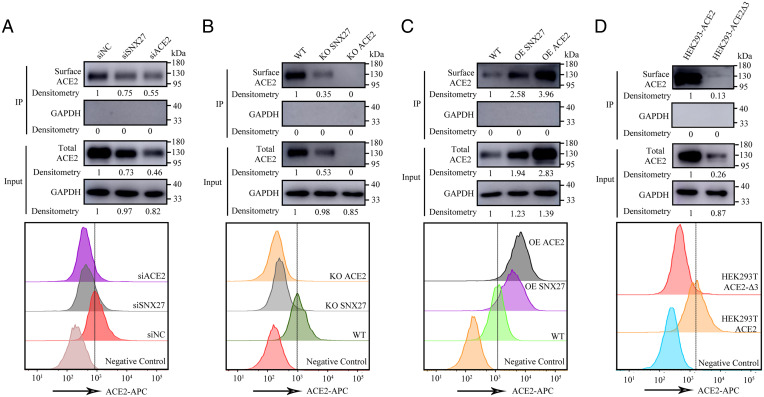
SNX27 regulates ACE2 protein cell surface homeostasis. (*A*) Surface and total expressions of receptor ACE2 were detected in siSNX27 and siACE2 Huh7 cells compared with siNC Huh7 cells. The plasma membrane proteins were biotin-labeled and immunoprecipitated by streptavidin beads for immunoblot analysis. Shown are representative histograms of flow cytometry analysis to determine cell surface expression of ACE2. (*B*) Surface and total expressions of receptor ACE2 were detected in SNX27 and ACE2 KO Huh7 cells compared with wild-type (WT) Huh7 cells. The plasma membrane proteins were biotin-labeled and immunoprecipitated by streptavidin beads for immunoblot analysis. Shown are representative histograms of flow cytometry analysis to determine cell surface expression of ACE2. (*C*) Surface and total expressions of receptor ACE2 were detected in SNX27 and ACE2 OE Huh7 cells compared with wild type (WT) Huh7 cells. The plasma membrane proteins were biotin-labeled and immunoprecipitated by streptavidin beads for Immunoblot analysis. Shown are representative histograms of flow cytometry analysis to determine cell surface expression of ACE2. (*D*) Surface and total expressions of receptor ACE2 were detected in HEK293T-ACE2 and HEK293T-ACE2Δ3 cells. The plasma membrane proteins were biotin-labeled and immunoprecipitated by streptavidin beads for immunoblot analysis. Shown are representative histograms of flow cytometry analysis to determine cell surface expression of ACE2. The dashed line indicates the gate between the negative and positive cells. The dots were quantified based on the densitometry measured by ImageJ, and the values are shown after normalization to the dot density of ACE2 or GAPDH.

Previous studies have already shown the function of SNX27 in regulating cell surface abundance of its cargoes (18). Therefore, we decided to test whether cellular homeostasis of ACE2 can be influenced by SNX27. Because human liver cancer cell line Huh7 has endogenous expression of ACE2, whereas the ACE2 expression could not be detected in HEK293T and HeLa cells (*SI Appendix*, Fig. S2), we examined the stability of ACE2 in Huh7 cell under the conditions of knockdown (KD), knockout (KO), and overexpression (OE) of SNX27. We used small interfering RNA (siRNA) and the lenti-CRISPR system to target SNX27 in Huh7. As for the KO of SNX27, we picked up a single colony, and the KO of SNX27 was confirmed by sequencing and Western blot. The biotin-labeled cell surface ACE2 was immunoprecipitated by Strep beads and determined by Western blot. Compared to the siRNA negative control (siNC), the depletion of SNX27 by RNA interfering decreased the total ACE2 protein level, which is similar to the case of KD of ACE2. The surface protein level of ACE2 also decreased when SNX27 was knocked down (Fig. 2*A*). Accordingly, both total and surface ACE2 protein levels decreased even further when SNX27 was knocked out in the Huh7 cell (Fig. 2*B*), which is consistent with the result shown by Zhu et al. (36). Conversely, compared to the ACE2 expression level in the parental Huh7 cell, the total and surface ACE2 protein levels increased when SNX27 was stably overexpressed (Fig. 2*C*). Then, we asked whether the correlation between ACE2 and SNX27 is due to their direct interaction. We examined the stability of ACE2 and ACE2 mutant with PBM deletion (ACE2Δ3) in stably transfected HEK293T cells in which the endogenous ACE2 expression is undetectable (*SI Appendix*, Fig. S2). The HEK293T cells were infected with the same multiplicity of infection (MOI) of wild-type ACE2 and ACE2 mutant lentiviruses, and the total and surface expressions of ACE2 were detected by Western blot. Indeed, the expression level of mutant ACE2Δ3 is much lower than the wild-type one, indicating SNX27 regulates the stability of ACE2 through PBM/PDZ interaction (Fig. 2*D*).

We also utilized flow cytometry to determine the cell surface expression level of ACE2 by disturbing the expression of SNX27. Anti-ACE2 antibody was used to measure the amount of ACE2 on the Huh7 cell surface. As shown by flow cytometry, siNC Huh7 cells are rich in ACE2 on their surface, whereas the amount of cell surface ACE2 in SNX27 KD cells diminished, resembling the scenario of KD of ACE2 (Fig. 2*A*). In the case of SNX27 KO cells, the surface expression level of ACE2 decreased even further and is close to the expression level of ACE2 KO cells (Fig. 2*B*). However, when SNX27 was overexpressed in Huh7 cells, the cell surface ACE2 expression level increased, as in the case of cells overexpressing ACE2 (Fig. 2*C*). We also examined the cell surface expression level of ACE2 mutant with PBM deletion (ACE2Δ3) in stably transfected HEK293T cells by flow cytometry. The result shows ACE2Δ3 has much less cell surface expression than the wild-type one (Fig. 2*D*). According to the result obtained above, our flow cytometry data are consistent with the result of the experiment of Strep beads pulldown assay. Taken together, SNX27 regulates the expression level of cell surface ACE2 by its direct PBM/PDZ interaction.

**Fig. 3.**
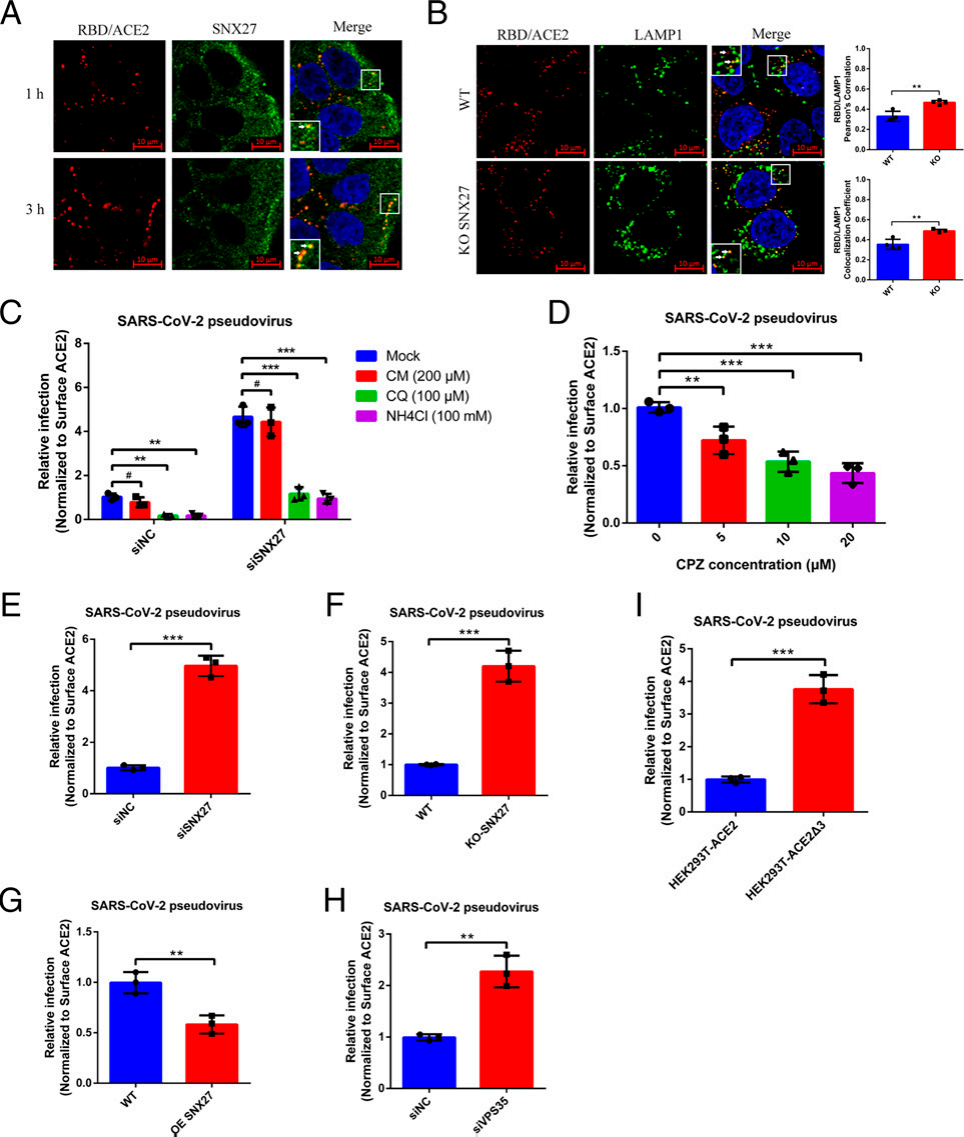
SNX27-retromer suppresses the SARS-CoV-2 pseudovirus infection. (*A*) Huh7 WT cells were treated with 1 µM RBD-AF555 and incubated at 37 °C for 1 and 3 h. Then, the cells were fixed and processed for indirect immunofluorescence using antibodies against SNX27 protein. Cell nuclei were counterstained with Hoechst 33342. (Scale bars, 10 μm.) *Insets* show enlarged field of colocalization of RBD/ACE2 and SNX27. (*B*) Huh7 WT and SNX27 KO cells were treated with 1 µM RBD-AF555 and incubated in the 37 °C for 1 h. Then, the cells were fixed and processed for indirect immunofluorescence using antibodies against LAMP1 protein. Cell nuclei were counterstained with Hoechst 33342. (Scale bars, 10 μm.) *Insets* show enlarged field of colocalization of RBD/ACE2 and LAMP1. (*C*) Huh7 siSNX27 and siNC cells were pretreated with 200 µM CM, 100 µM CQ, and 100 mM NH_4_Cl for 3 h before SARS-CoV-2 pseudovirus infection. After 24 hpi, the cells were lysed, and the infectivities of the pseudoviruses are represented as luciferase activities. (*D*) Huh7 WT cells were pretreated with indicated concentrations of CPZ for 3 h before SARS-CoV-2 pseudovirus infection. After 24 hpi, the cells were lysed, and the infectivities of the pseudoviruses are represented as luciferase activities. (*E*) Huh7 siSNX27 and siNC cells were infected with SARS-CoV-2 pseudovirus for 24 h. The cells were lysed, and the infectivities of the pseudoviruses are represented as luciferase activities. (*F*) Huh7 WT and SNX27 KO cells were infected with SARS-CoV-2 pseudovirus for 24 h. The cells were lysed, and the infectivities of the pseudoviruses are represented as luciferase activities. (*G*) Huh7 WT and SNX27 OE cells were infected with SARS-CoV-2 pseudovirus for 24 h. The cells were lysed, and the infectivities of the pseudoviruses are represented as luciferase activities. (*H*) Huh7 siVPS35 and siNC cells were infected with SARS-CoV-2 pseudovirus for 24 h. The cells were lysed, and the infectivities of the pseudoviruses are represented as luciferase activities. (*I*) HEK293T-ACE2 and HEK293T-ACE2Δ3 cells were also infected with SARS-CoV-2 pseudovirus for 24 h. The cells were lysed, and the infectivities of the pseudoviruses are represented as luciferase activities. The luciferase activities were normalized to the surface ACE2 MFI level by flow cytometry analysis. The data represent the mean ± SD of three independent experiments. *P < 0.05, **P < 0.01, ***P < 0.001, ^#^*P* > 0.05. *Insets* show enlarged field of colocalization of RBD/ACE2 and SNX27.

Next, we performed the immunofluorescence experiment to check the colocalization of SNX27 and ACE2 in the cell and determine the trafficking route used by ACE2. Because ligand stimulation can induce the endocytosis of ACE2, we carried out this experiment by using RBD treatment. In order to exclude the possibility of nonspecific cell surface binding of RBD, we stained both wild-type and ACE2 KO Huh7 cells by fluorophore-labeled RBD. However, only the wild-type one can be stained (*SI Appendix*, Fig. S3), suggesting the RBD staining relies on ACE2 receptor, and no other RBD binder exists on the cell surface. We also stained other ACE2 free cells such as HEK293T and HeLa cells, and they cannot be stained by RBD (*SI Appendix*, Fig. S3), which further confirms the specificity of RBD staining. Therefore, we utilized fluorophore-labeled RBD as a proxy of ACE2 to check the travel of ACE2 in the cell. One micromole RBD protein was incubated with Huh7 cells on the ice for 30 min, and then the extra RBD protein was washed away, and finally chased at different time points (1 and 3 h) at 37 °C. The internalized ACE2 was shown to be colocalized with early endosome (sorting endosome) maker Rab5 (*SI Appendix*, Fig. S4). The apparent colocalization of SNX27 and ACE2 can be observed even after 1 h of treatment, and colocalization increased after 3 h of treatment (Fig. 3*A*), which is consistent with the result of enhanced binding between ACE2 and SNX27 after RBD treatment in the co-IP experiment (Fig. 1*E*). There are two SNX27-mediated recycling pathways (fast and slow) for EE-localized receptors, and the small GTPase Rab4 and Rab11 are markers for fast and slow recycling endosomes, respectively (42–44). In order to figure out which pathway is adopted by ACE2 recycling, we performed colocalization assay between ACE2 and Rab4 or Rab11 in Huh7 cells. The confocal microscopy result shows internalized ACE2 colocalized with Rab4 but not with Rab11, suggesting ACE2 receptor undergoes fast recycling through SNX27 (*SI Appendix*, Fig. S4). To further unravel the mechanism of SNX27 regulating ACE2 trafficking, colocalization of ACE2 with the lysosomal marker LAMP1 was analyzed using SNX27 KO Huh7 cells. After RBD protein treatment in control cells, we can see the colocalization of ACE2 and LAMP1, but there are still lots of ACE2 foci which didn’t colocalize with LAMP1 (Fig. 3B). However, the colocalization of ACE2 and LAMP1 increased in SNX27 KO Huh7 cells. We utilized the colocalization correlation coefficient and Pearson’s correlation coefficient to quantitatively measure the colocalization of these two proteins. Both methods show the same quantification results of increased colocalization in the SNX27 KO Huh7 cells compared with the control cells (Fig. 3B). Thus, these data support the idea that internalized ACE2 receptor was sorted to the recycling pathway via SNX27, avoiding lysosome/late endosome degradation.

### SARS-CoV-2 Uses a Clathrin-Dependent Endocytosis Pathway for Its Entry in Huh7 Cells.

Since we have established the link between ACE2 and SNX27, it is intriguing to know how the interaction between ACE2 and SNX27 affects viral infection. First, we asked whether ACE2-mediated viral endocytosis plays an essential role in SARS-CoV-2 entry. Previous studies on SARS-CoV show that it mainly uses the endocytic pathway for entry, although there are two controversial endocytic mechanisms proposed (clathrin-dependent endocytosis and clathrin-independent endocytosis) (45–47). As for SARS-CoV-2, it is reported that SARS-CoV-2 enters a cell through two pathways: the early route, by directly fusing with plasma membrane, and late route, by receptor-mediated endocytosis (11, 13, 48). To figure out the exact route used by SARS-CoV-2 in Huh7 cells, we exploited lentivirus pseudotyped by spike protein from SARS-CoV-2 to infect Huh7 cells and evaluated the infection efficacy by measuring the luciferase activity after invasion. On the other hand, TMPRSS2 protease is considered a major enzyme responsible for early pathway viral entry which can be blocked by Camostat mesylate (11). We first confirmed the activity of Camostat mesylate by inhibiting the activity of trypsin enzyme (*SI Appendix*, Fig. S5). Then, we added Camostat mesylate to culture medium and utilized SARS-CoV-2 pseudovirus to infect the Huh7 cells. Surprisingly, there is no significant difference between drug treatment and mock, although there is endogenous TMPRSS2 expression in Huh7 cells (49) (Fig. 3C and *SI Appendix*, Fig. S2), which suggests SARS-CoV-2 pseudovirus does not use the early pathway for its entry in Huh7 cells. Then, we used ammonium chloride and chloroquine, lysosomotropic agents, to examine the second pathway SARS-CoV-2 pseudovirus may utilize. As expected, both of them can efficiently block pseudovirus entry (Fig. 3C and *SI Appendix*, Fig. S6*A*), which supports the notion of endocytosis-dependent viral entry. Furthermore, we used compound chlorpromazine (CPZ), the inhibitor of AP2 (50), to determine whether endocytosis is clathrin dependent. The infection was inhibited in a dose-dependent manner, and, when the concentration of chlorpromazine reached 20 μM, the infection was reduced by 50% (Fig. 3D and *SI Appendix*, Fig. S6*B*), which indicates the clathrin-dependent endocytosis pathway plays an important role during SARS-CoV-2 pseudovirus entry, consistent with the recent study performed in HEK293T cells (48).

### SNX27/ACE2 Regulates the SARS-CoV-2 Infection by Preventing It from Entering Lysosome/Late Endosome.

**Fig. 4.**
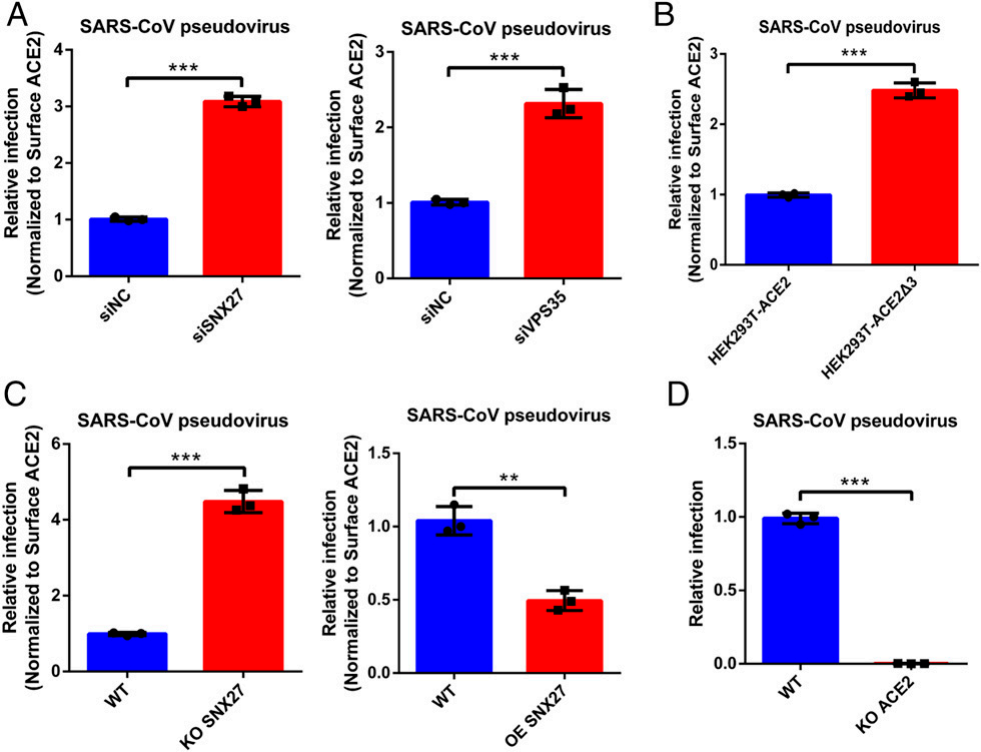
The SNX27-retromer suppresses the SARS-CoV pseudoviruses infection. (*A*) Huh7 siSNX27, siVPS35, and siNC cells were infected with SARS-CoV pseudovirus for 24 h. The cells were lysed, and the infectivities of the pseudoviruses are represented as luciferase activities. (*B*) HEK293T-ACE2 and HEK293T-ACE2Δ3 cells were also infected with SARS-CoV pseudovirus for 24 h. The cells were lysed, and the infectivities of the pseudoviruses are represented as luciferase activities. (*C* and *D*) Huh7 WT, ACE2 KO, SNX27 KO, and SNX27 OE cells were infected with SARS-CoV pseudovirus for 24 h. The cells were lysed, and the infectivities of the pseudoviruses are represented as luciferase activities. The luciferase activities were normalized to the surface ACE2 MFI level by flow cytometry analysis. The data represent the mean ± SD of three independent experiments. **P < 0.01, ***P < 0.001.

Because SNX27 could sort ACE2/RBD complex into the recycling pathway, we asked whether endocytic ACE2/virus could be regulated by SNX27 as well. We next examined the infection by disturbing the expression level of SNX27. Given that the amount of ACE2 on the cell surface is positively correlated to the viral infection whether it is in the scenario of pseudovirus or authentic virus (*SI Appendix*, Fig. S7), and the cell surface protein level of ACE2 is tightly controlled by SNX27 (Fig. 2), we therefore normalized the cell surface protein level of ACE2 in order to eliminate the variation of ACE2 resulting from the SNX27 disturbance. The infection results were analyzed and discussed according to the results after surface ACE2 normalization, unless otherwise stated. The fold change of ACE2 mean fluorescence intensity (MFI) in flow cytometry was used during data preparation (*SI Appendix*, Fig. S8). Huh7 cells were transfected with siRNA targeting SNX27, and SARS-CoV-2 pseudovirus infection was carried out 48 h later. Compared to the siNC, the depletion of SNX27 enhanced the SARS-CoV-2 pseudovirus infection (Fig. 3E and *SI Appendix*, Fig. S9*A*), whereas the KD of ACE2 dramatically reduced the infection (*SI Appendix*, Fig. S9*A*). However, the infection promoted by SNX27 depletion is dramatically blocked by lysosomotropic agents (e.g., ammonium chloride and chloroquine) but not the early pathway inhibitor Camostat mesylate (Fig. 3C), suggesting more ACE2/virus complex reached lysosome/late endosome with the depletion of SNX27, and SNX27 diminishes infection by preventing viral lysosome/late endosome entry (Fig. 3E). Similarly, the KO of SNX27 also facilitates the pseudovirus infection (Fig. 3F and *SI Appendix*, Fig. S9*B*). Regarding the OE of SNX27, the pseudovirus infection efficacy was almost decreased by half after surface ACE2 normalization (Fig. 3G and *SI Appendix*, Fig. S9*B*). Next, we asked whether SNX27 functions through retromer complex, and we performed RNA interference (RNAi) to knock down VPS35, one subunit of retromer core component. Indeed, depletion of VPS35 also contributed the increased infection of SARS-CoV-2 pseudovirus, although the effect of VPS35 KD is less than that of SNX27 KD (Fig. 3H and *SI Appendix*, Fig. S9*A*). Thus, SNX27 and retromer worked together to inhibit the entry of pseudovirus. Because ACE2 uses its C-terminal PBM to engage with SNX27, we asked whether ACE2Δ3 disrupting the interaction with SNX27 exhibits the same behavior as the depletion of SNX27 during viral infection. We used SARS-CoV-2 pseudovirus to infect HEK293T cells stably expressing ACE2Δ3 or full-length ACE2. Similar to the results of SNX27 depletion, ACE2Δ3 promotes the infection of pseudovirus as well (Fig. 3I and *SI Appendix*, Fig. S9*B*). Additionally, as a SARS-CoV-2 closely related coronavirus, SARS-CoV uses the same receptor ACE2 for entry, and it is reasonable to consider that SNX27-retromer complex plays a pretty similar role during SARS-CoV entry. Indeed, when we used SARS-CoV pseudovirus to study the relationship between entry and SNX27-retromer complex, we found that the depletion of SNX27 or VPS35 and disrupting the interaction between SNX27 and ACE2 could promote SARS-CoV pseudovirus entry (Fig. 4 A and B, *SI Appendix*, Fig. S10 *A* and *B*), whereas OE of SNX27 leads to the opposite effect (Fig. 4C and *SI Appendix*, Fig. S10*C*), which is the same as the case of KO ACE2 (Fig. 4D). Therefore, SNX27-retromer complex employs a universal mechanism to regulate the infection of SARS coronaviruses that use ACE2 as a common receptor.

**Fig. 5.**
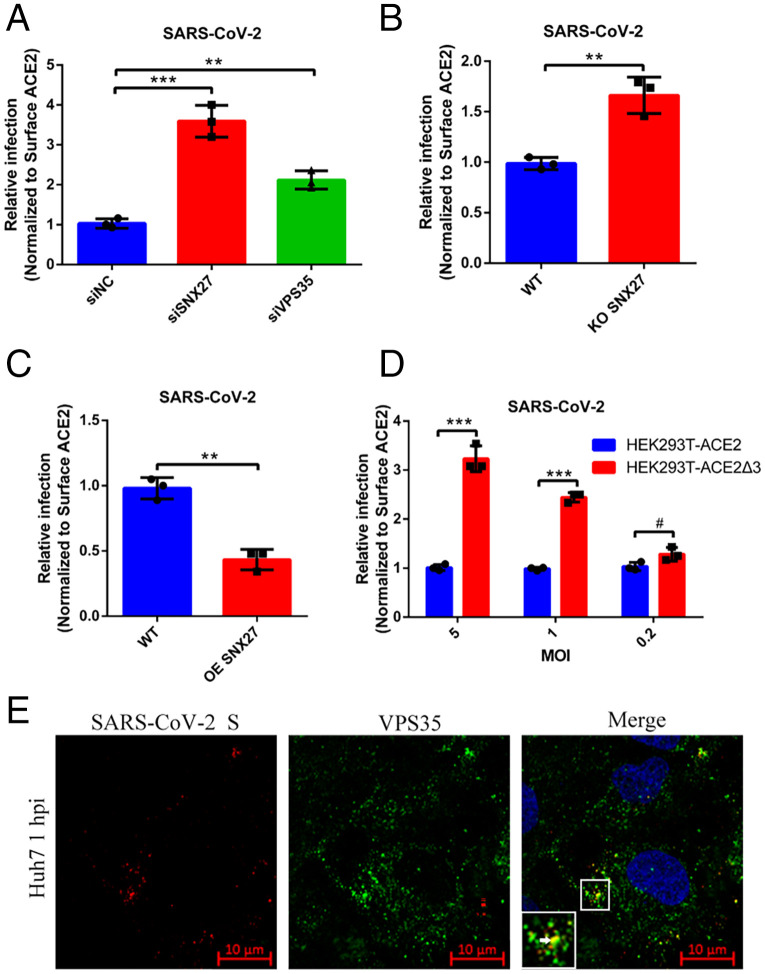
The SNX27-retromer suppresses authentic SARS-CoV-2 virus infection. (*A*) Huh7 siSNX27, siVPS35, and siNC cells were infected with SARS-CoV-2 at an MOI of 1. The relative concentration of viral RNA present in the supernatant at 16 hpi was determined by real-time qPCR analysis. (*B*) Huh7 WT and SNX27 KO cells were infected with SARS-CoV-2 at an MOI of 1. The relative concentration of viral RNA present in the supernatant at 16 hpi was determined by real-time qPCR analysis. (*C*) Huh7 WT and SNX27 OE cells were infected with SARS-CoV-2 at an MOI of 1. The relative concentration of viral RNA present in the supernatant at 16 hpi was determined by real-time qPCR analysis. (*D*) HEK293T-ACE2 and HEK293T-ACE2Δ3 cells were infected with SARS-CoV-2 (MOI = 5, 1, 0.2) for 16 h. The relative concentration of viral RNA present in the supernatant was determined by real-time qPCR analysis. The concentration of viral RNA was normalized to the surface ACE2 MFI level by flow cytometry analysis. (*E*) Huh7 WT cells were infected with SARS-CoV-2 (MOI = 10) and incubated in the 37 °C for 1 h. Then, the cells were fixed and processed for indirect immunofluorescence using antibodies against SARS-CoV-2 S and VPS35 protein. Cell nuclei were counterstained with Hoechst 33342. (Scale bars, 10 μm.) The data represent the mean ± SD of three independent experiments. **P < 0.01, ***P < 0.001, ^#^*P* > 0.05.

We next used authentic SARS-CoV-2 virus to evaluate the infection efficiency when SNX27-retromer complex was disturbed. We first performed the RNAi to silence SNX27 and VPS35 expression, and then Huh7 cells were infected with SARS-CoV-2 virus at an MOI of 1. We used qRT-PCR to detect ORF1 transcription of virus at 16 h postinfection (hpi), and the surface expression level of ACE2 was normalized (*SI Appendix*, Fig. S8). Depletion of both SNX27 and VPS35 by RNAi could promote the replication of SARS-CoV-2 (Fig. 5A and *SI Appendix*, Fig. S11*A*), which is consistent with our pseudovirus infection results. Then, we used the SARS-CoV-2 virus to infect the SNX27 KO Huh7 cells for 16 h, and infection  increased as expected after surface ACE2 normalization (Fig. 5B and *SI Appendix*, Fig. S11*B*). We also performed the SARS-CoV-2 infection using Huh7 cells with lentiviral OE of SNX27. Through normalization of surface ACE2, the relative infection in SNX27 OE cells was reduced (Fig. 5C and *SI Appendix*, Fig. S11*B*). Next, we used HEK293T cells stably expressing ACE2 and mutant ACE2Δ3 to determine viral infectivity. We used a series of titers of viruses (MOIs: 0.2, 1, 5) to infect HEK293T cells and measured the replication at 16 hpi. We found that the relative infection in ACE2Δ3-expressing cells increased dramatically with the higher viral titers (1, 5), compared with ACE2-expressing cells after surface ACE2 normalization (Fig. 5D and *SI Appendix*, Fig. S11*B*). Additionally, we performed immunofluorescence assay to determine whether SARS-CoV-2 virion can be recruited to the SNX27-retromer recycling machinery. Huh7 cells were infected with SARS-CoV-2 at an MOI of 10 and collected at 1 hpi. The viruses and retromer complex were detected by spike protein antibody and VPS35 antibody, respectively. We can detect the precise colocalization of SARS-CoV-2 and retromer complex immediately after the virus invasion (Fig. 5E). Taken together, our data suggest the cell entry of SARS-CoV-2 is regulated by SNX27-retromer complex via interacting with the PBM of ACE2.

**Fig. 6.**
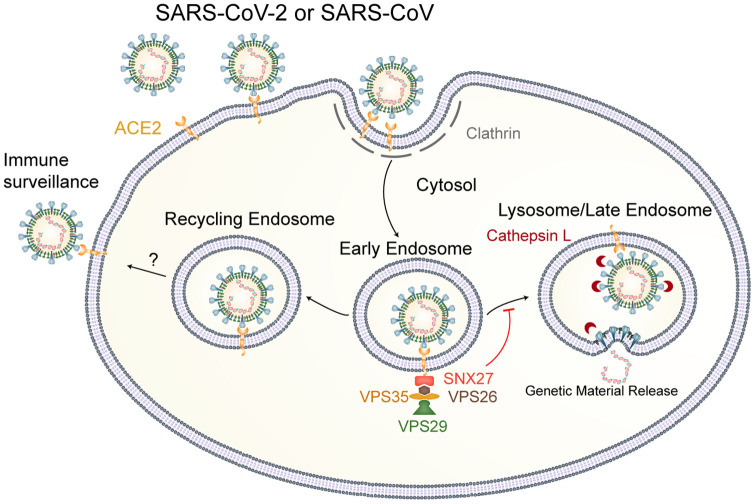
Proposed model of SNX27 regulating SARS-CoV-2 entry. When SARS-CoV-2 enters the cell through the clathrin-dependent endocytic pathway, it can be directed by the SNX27-retromer complex to recycling endosome and avoid lysosome/late endosome entry, resulting in decreased infection.

## Discussion

In the present study, our structural model of the ACE2-PBM/SNX27-PDZ complex demonstrates that ACE2 possessing an unusual PDZ ligand of SNX27 which lacks of acidic residues in position P_-3_, the determinant of strong binding to SNX27 (38), could also engage with SNX27 strongly via extra hydrogen bonds and a larger hydrophobic C-terminal residue. We show that internalized ACE2 can be sorted by SNX27 through PBM/PDZ interaction at an EE. SNX27 directs ACE2 to the Rab4-positive recycling endosome, preventing it from entering the lysosome/late endosome degradation pathway. Therefore, SNX27 plays an essential role in regulating the cell surface homeostasis of ACE2 under physiological conditions. We also demonstrated that the entry of SARS-CoV-2 pseudovirus uses the late route in a clathrin-dependent manner in Huh7 cells. Furthermore, we found that SNX27-retromer could inhibit the entry of SARS-CoV-2 by preventing ACE2/virus from entering lysosome/late endosome when the late pathway prevails.

Given that ACE2/RBD complex is stable under the acidic environment as shown by gel filtration results (*SI Appendix*, Fig. S12), the low pH at an EE probably cannot dissociate the virus from ACE2, resulting in the virus being sorted as the cargo of SNX27 bridged by ACE2. To date, almost all of SNX27 cargoes identified are transmembrane-spanning proteins (18), and these protein cargoes can be sorted into a tubular structure with a diameter of 20 nm to 50 nm decorated by SNX-BAR-retromer complex (22). Due to the large size of the ACE2/virus complex (about 90 nm in diameter of SARS-CoV-2 virion) (51), it is still an open question how the viral cargo fits into endosomal sorting tubules. According to previous electron microscopy studies on the architecture of SARS-CoV-2 (51–53), viral particles are plastic and versatile, which is likely to make the sorting possible. After it is sorted to recycling endosome, the ACE2/virus complex may travel back to the cell surface, making the entry nonproductive, and the cell surface exposure of virus is more vulnerable to immune surveillance (54). Additionally, if it were recycled back to the plasma membrane, cell surface–residing ACE2 sheddase like ADAM17 may have more opportunities to process membrane-bound ACE2 to soluble form, and peels ACE2/virus off from the plasma membrane (55). The itineraries of internalized virus can affect their infectivity (43). A virus like foot and mouth disease virus which is sensitive to acidic environments can be sorted to recycling endosome, avoiding lysosome/late endosome degradation (56). In contrast, the sorting of SARS-CoV-2 to recycling endosome will decrease its lysosome/late endosome–mediating entry. In such a case, SNX27-retromer can be considered as a type of antiviral defense line of the host innate immune system. Based on our data, we proposed a model for the SARS-CoV-2 infection regulated by SNX27-retromer complex. In the scenario of the late pathway, the attached virus (SARS-CoV or SARS-CoV-2) could undergo receptor-mediated endocytosis and the sorting of virus by SNX27-retromer at an EE reduced viral lysosome/late endosome entry (Fig. 6).

As for the virus using the cell surface entry pathway only, trafficking to lysosome/late endosome will not be implicated, which results in the amount of surface ACE2 being the determinant of entry. Considering the regulation of ACE2 by SNX27, SNX27 could recycle more ACE2 to the cell surface, causing cell susceptibility to SARS-CoV-2, and SNX27 may play a proviral role during infection. However, the entry of SARS-CoV-2 is largely cell type dependent, and it is still a matter of debate (12). Therefore, SNX27, together with the entry pathway, may affect the tropism of SARS-CoV-2 and the outcome of COVID-19. The versatile issue of proviral and antiviral entry regulated by SNX27 should be addressed further in the future.

All in all, we identified SNX27-retromer complex as a host factor for the entry of SARS-CoV-2; it is important for us to employ this pathway to develop new antiviral therapies.

## Materials and Methods

### Antibodies and Reagents.

The following antibodies were used in this study: anti-ACE2 (Abcam, ab15348), anti-SNX27 (Abcam, ab77799), anti-VPS35 (Proteintech, 10236-1-AP), anti-GAPDH (Proteintech, 60004-1-Ig), anti-SARS-CoV-2 Spike (GeneTex, GTX135356), anti-LAMP1 (Cell Signaling Technology, 15665S), anti-HA (Cell Signaling Technology, 3724), anti-FLAG (Sigma-Aldrich, F1804), and anti-TMPRSS2 Antibody (Santa Cruz, sc-515727). Secondary antibodies are as follows: HRP-conjugated goat anti-mouse IgG (Sigma-Aldrich, A9917), HRP-conjugated goat anti-rabbit IgG (Sigma-Aldrich, A0545), Goat anti-Rabbit IgG (H+L) Highly Cross-Adsorbed Secondary Antibody, Alexa Fluor Plus 488 (Invitrogen, A32731), Goat anti-Mouse IgG (H+L) Highly Cross-Adsorbed Secondary Antibody, and Alexa Fluor Plus 488 (Invitrogen, A32723). Chemicals and reagents are as follows: Camostat mesylate (CM, MedChemExpress, HY-13512), chloroquine (CQ, Sigma-Aldrich, C6628), NH_4_Cl (Sigma-Aldrich, V900222), and chlorpromazine (CPZ, Sigma-Aldrich, C0982).

### Cell Lines and Virus.

HEK293T (ATCC, CRL-3216) cells and Huh7 (Institute of Basic Medical Sciences CAMS, 3111C0001CCC000679) cells were grown in Dulbecco’s modified Eagle’s medium (DMEM) (Gibco, C11995500BT) supplemented with 10% fetal bovine serum (FBS; Gibco, 10270-106, 10437-028), 100 IU/mL penicillin, and 10 μg/mL streptomycin (Gibco, 15140-122) at 37 °C in 5% CO_2_. SARS-CoV-2 pseudovirus was kindly provided by National Institutes for Food and Drug Control. SARS-CoV-2 named BetaCoV/Wuhan/IVDC-HB-envF13/2020 (Accession ID: EPI_ISL_408511) was isolated by National Institute for Virus Disease Control and Prevention, Chinese Center for Disease Control and Prevention. Vero E6 (ATCC, CRL-1586) cells were applied to the reproduction of SARS-CoV-2 stocks. All the infection experiments were performed in a biosafety level-3 laboratory.

### Protein Expression and Purification.

The coding region for the PDZ domain of SNX27 (residues 40 to 135) was subcloned into a pET-21b (Novagen) vector generating a construct with His6-tag at its C terminus. RBD (residues 331 to 531) of S protein from SARS-CoV-2 and the extracellular domain of human ACE2 (residues 17 to 741) fused with alkaline phosphatase signal peptide at the N terminus and a His8-tag at the C terminus were subcloned into pcDNA3.1(+) vector (Invitrogen). PDZ domain protein was overexpressed in *Escherichia coli* BL21 (DE3) and purified by nickel affinity chromatography. The protein was further purified by gel filtration chromatography and concentrated to 10 mg/mL for crystallization and 20 mg/mL ITC assay. RBD (residues 331 to 531) and ACE2 (residues 18 to 741) were overexpressed in FreeStyle 293-F cells (Invitrogen) as secreted proteins, and then they were purified by nickel affinity chromatography and gel filtration chromatography. For the fluorophore-labeling of RBD, RBD in Hepes buffer at 10 mg/mL was incubated with amine-reactive Alexa Fluor-555(Invitrogen) for 1 h at room temperature. RBD conjugated with Alexa Fluor-555 was further purified by gel filtration chromatography.

### Gel Filtration Assay.

The RBD/ACE2 complex dissociation was examined under various pH conditions using a superdex200 10 × 100 column (GE healthcare). The buffers used are as follows: 20 mM NaAC, 150 mM NaCl, pH 4.5; 20 mM NaAC, 150 mM NaCl, pH 5.5; 20 mM MES, 150 mM NaCl, pH 6.5; and 20 mM Tris, 150 mM NaCl, pH 7.5. The complex was obtained by mixing RBD and ACE2 protein at room temperature for 1 h. Then, it was directly applied to the gel filtration column, and the protein was detected by sodium dodecyl sulfate polyacrylamide gel electrophoresis (SDS-PAGE) and Coomassie blue staining.

### Generation of Stable Lentiviral Cell Lines.

The genes of ACE2 (GenBank accession number: NM_021804.1), SNX27 (GenBank accession number: NM_001330723.2), Rab4a (GenBank accession number: NM_004578.4), Rab5 (GenBank accession number: M28215.1), and Rab11 (GenBank accession number: X56740.1) were subcloned into the lentiviral vector pTY (gift from Hui Yang, Fudan University, Shanghai, China) for the generation of lentiviral particles. Lentiviral particles were produced and harvested in HEK293T cells. Huh7 and HEK293T cells were transduced with lentiviral particles to produce stably expressing cell lines. Following transduction, target protein-expressing cells were selected with hygromycin accordingly. To generate an ACE2 and SNX27 KO Huh7 cell line, the ACE2 single guide RNA (sgRNA: TGCTGCTCAGTCCACCATTG) and SNX27 sgRNA (GTGTGTTCAATACGAGTAAT) were cloned into LentiCRISPRv2. The sgRNA was transfected into Huh7 cells; 48 h later, transfected cells were selected with puromycin. Selected cells were plated into 96-well plates to seed single cell colonies. After 3 weeks or 4 weeks, colonies were expanded and lysed, and KO was validated by immunoblotting for ACE2 and SNX27.

### Viral Infection and Cell Treatment.

According to the requirements of the different experiments, cells were seeded in 48-well plates (2.5 × 10^4^ cells per well) and infected with SARS-CoV-2 pseudovirus. After 24-h incubation, cell lysates were transferred into luminometer plates (Microfluor 96-well plates). Luciferase substrate, included in luciferase assay system (Promega, E1501), was added. Luciferase activity was measured using a GloMax 96 Microplate Luminometer. For analysis of the drug inhibition experiment, Huh7 cells were pretreated with chloroquine (100 μM), Camostat mesylate (100 μM), and NH_4_Cl (100 mM) for 3 h and infected with SARS-CoV-2 pseudovirus for 24 h. For SARS-CoV-2 experiments, Huh7 cells were seeded in 24-well plates (1 × 10^5^ cells per well) and infected with SARS-CoV-2 at an MOI of 1. HEK293T-ACE2 cells were seeded in 24-well plates (1 × 10^5^ cells per well) and infected with SARS-CoV-2 at an MOI of 0.2, 1, and 5. After 1-h incubation at 37 °C, the unbound viruses were removed by washing for three times with phosphate-buffered saline (PBS); then, the cells were cultured in DMEM supplemented with 2% FBS at 37 °C for 16 h.

### Transfection and Gene Silencing with siRNAs.

SMARTpool siRNAs targeting ACE2 (5′-GCGACUUCAGGAUCCUUAU-3′), SNX27 (5′-CGGUUACAGUCAGGGUUAA-3′), and VPS35 (5′-GCUGGCAGAAUUGCCCUUA-3′) were designed and synthesized by the supplier GenePharma Biotechnology. We selected the most efficient siRNA for each gene for the experiment. Huh7 cells were seeded in 48-well plates (2.5 × 10^4^ cells per well) and transfected with 300 nM siRNA using jetPRIME in vitro DNA & siRNA Transfection Reagent (Polyplus Transfection, 114). Then, the cells were incubated in DMEM at 37 °C for 48 h or 72 h. The reaction mixture was discarded, and the cells were infected with SARS-CoV-2 pseudovirus and detected by Luciferase Assay System. A control siRNA (UUCUCCGAACGUGUCACGU) was maintained in our laboratory and used as a negative control. The silencing efficiency was measured by immunoblotting.

### Immunoprecipitation and Immunoblotting Analysis.

The ACE2 gene with N-terminal Flag tag was cloned into the pCMV vector. ACE2Δ3 has a stop codon at amino acid 802, and ACE2Δ34 has a stop codon at amino acid 771. The SNX27 gene with C-terminal HA tag was cloned into the pcDNA3.1(+). HEK293T was seeded in six-well plates (1 × 10^6^ cells per well) and cotransfected with pCMV-ACE2, Δ3, Δ34, and pcDNA3.1(+)-SNX27 for 48 h and stimulated by 1 μM RBD protein, then incubated on ice with immunoprecipitation lysis buffer (Beyotime, P0013). For each sample, 500 μL of lysate was incubated with 5 μL of ANTI-FLAG M2 Magnetic Beads for 2 h at room temperature. The resin beads were washed with TBS buffer to remove all the nonspecifically bound proteins. The precipitates were detected by SDS-PAGE and immunoblotting. The immunoblotting analysis method was based on our previous study (57). Briefly, the samples were separated by SDS-PAGE and transferred to 0.22-µm polyvinylidene difluoride membranes (Millipore, ISEQ00010). The membrane was incubated with the primary antibody and then with the HRP-conjugated secondary antibody and finally detected by ECL kit (Millipore, WBKLS0500). Images were obtained with Armersham Image 600 (GE Healthcare), and the intensity of blots was analyzed with ImageJ software (NIH).

### Biotinylation of Plasma Membrane Proteins.

The biotinylation of ACE2 method was adapted from a previous report (36). Briefly, Huh7 cells seeded in six-well plates 24 h prior to experiment were chilled on ice for 30 min, and labeled with 2.5 mg/mL biotin (Thermo Fisher, 21331) for 1 h. After washing with PBS, cells were lysed in radioimmunoprecipitation assay (RIPA) buffer (Beyotime, P0013) in the presence of a mixture of protease inhibitors (Sigma-Aldrich, P8340), and immunoprecipitated with Streptavidin agarose beads (Sigma, 88817) overnight at 4 °C. Beads were then washed three times with RIPA buffer. The samples were eluted into 5× loading buffer and boiled for immunoblotting analysis as described above. The unimmnoprecipitated lysates were used as a loading control.

### Confocal Immunofluorescence Microscopy.

Cells were seeded in glass-bottomed cell culture dishes (NEST, 20 mm, 4 × 10^5^ cells per dish). For SARS-CoV-2 entry experiments, Huh7 cells were seeded in 24-well plates (1 × 10^5^ cells per well) and infected with SARS-CoV-2 at an MOI of 10. For the colocalization experiments, Huh7 cells were treated with 1 µM RBD labeled with Alexa Fluor 555 (RBD-AF555) at the indicated time point. The cells were washed three times with PBS and treated with 0.1% Triton X-100 (Solarbio, T8200) for 15 min. Then, the cells were incubated with 1% bovine serum albumin (Sigma-Aldrich, A7906) and the appropriate primary antibodies for 2 h at 37 °C before being washed and incubated simultaneously with fluorescein isothiocyanate– or tetramethylrhodamine-conjugated secondary antibodies. Finally, the cells were treated with a Hoechst 33342 (Sigma-Aldrich, B2261) solution for 3 min and analyzed under a confocal microscope (CLSM; Leica SP8).

### Flow Cytometry Analysis.

Huh7 cells were seeded in 12-well plates (2 × 10^5^ cells per well) and treated with ACE2 antibody labeled with Alexa Fluor 647 (ACE2-AF647) at 4 °C for 1 h. After adsorption, the medium was discarded, and the cells were subsequently washed with PBS. Then, cells were cultured in maintenance medium containing 2% serum at 37 °C 30 min. After washing, the cells were analyzed using a BD LSRFortessa X-20 Special Order System. All the above data were analyzed using FlowJo.

### Quantification of Viral RNA.

Cells were seeded in 24-well plates (1 × 10^5^ cells per well) and infected with SARS-CoV-2. After 1 h incubation at 37 °C, the cells were cultured in DMEM supplemented with 2% FBS at 37 °C for 16 h. SARS-CoV-2 detection was performed using the One Step PrimeScript RT-PCR kit (TaKaRa, RR064A) on the Applied Biosystems QuantStudio 7 Real-Time PCR System (Thermo Fisher Scientific). Culture supernatant was harvested at 16 h, and viral titer was tested by using qRT-PCR (forward primer: CCCTGTGGGTTTTACACTTAA; reverse primer: ACGATTGTGCATCAGCTGA; probe: 5′-FAM-CCGTCTGCGGTATGTGGAAAGGTTATGG-BHQ1-3′). The Ct value was changed to viral relative infection by formula 2^−△CT^.

### ITC Assay.

ITC measurement was performed, to test the binding of ACE2-PBM peptide to the PDZ domain of SNX27, with the MicroCal iTC200 (GE Healthcare) at 25 °C. ACE2-PBM peptide was dissolved in DMSO and diluted in the protein buffer (20 mM Tris pH 8.0; 150 mM NaCl) to a final concentration of 125 µM with 5% DMSO. The ACE2-PBM peptide was added to the syringe. PDZ protein was diluted in the protein buffer to a final concentration of 100 µM with 5% DMSO to avoid buffer mismatch and then was added to the sample cell. A control titration of buffer–buffer and ACE2-PBM peptide–buffer was performed according to the same protocol.

### Crystallization and Structure Determination.

Crystallization trials were performed in 48-well plates using the sitting-drop vapor diffusion method at 16 °C. For the crystallization of PDZ/ACE2-PBM (-DDVQTSF-COO^−^) complex, PDZ and PBM peptide (synthesized by Beijing SciLight Biotechnology Ltd. Co.) were mixed at an equimolar ratio and directly subjected to crystallization screens. The crystals grew in the condition containing 0.2 M sodium sulfate, 20% wt/vol polyethylene glycol 3350. Crystallization buffer supplemented with 25% (vol/vol) glycerol was used as the cryoprotectant. The crystals were flash cooled in liquid nitrogen before data collection. Data collection was carried out at 100 K on beamline BL02U1 at the Shanghai Synchrotron Radiation Facility (SSRF). The dataset was processed by using the HKL2000 software package (58). The structure was solved by molecular replacement (59) using the Phaser program (60). The PDZ structure of SNX27 [Protein Data Bank (PDB) ID: 4Z8J] was used as the search model to obtain the initial phase of the complex. The ACE2-PBM peptide was manually built into the map using the program Coot (61), and the structure was refined using phenix.refine in Phenix (62). Data collection and structure refinement statistics of PDZ/ACE2-PBM complex are listed in *SI Appendix*, Table S1. The coordinates of PDZ/ACE2-PBM complex were deposited in the PDB repository, ID 7E0B. Model quality was evaluated by using MolProbity (63). All structural figures were rendered using Pymol software (http://www.pymol.org/2).

### Statistical Analysis.

The data are expressed as the mean ± SD. The significance of the variability between the different treatment groups was calculated with the two-tailed, unpaired Student *t* test or two-way ANOVA followed by Tukey’s multiple comparisons test using GraphPad Prism 6.0 software (Graph Pad Software Inc.). A *P* value of <0.05 was considered to indicate statistical significance.

## Supplementary Material

Supplementary File

## Data Availability

The coordinates of PDZ/ACE2-PBM complex were deposited in the PDB repository, https://www.rcsb.org/ (PDB ID 7E0B). All other study data are included in the article and/or *SI Appendix*.
