# Supplementary material for: SNX27 suppresses SARS-CoV-2 infection by inhibiting viral lysosome/late endosome entry
Source: Proc Natl Acad Sci U S A. 2022 Jan 13;119(4):e2117576119. doi: 10.1073/pnas.2117576119 (PMC8794821; doi:10.1073/pnas.2117576119)
Supplement: Supplementary File [file pnas.2117576119.sapp.pdf]

Supplementary Information for

SNX27 suppresses SARS-CoV-2 infection by inhibiting viral lysosome/late endosome entry

Bo Yang, Yuanyuan Jia, Yumin Meng, Ying Xue, Kefang Liu, Yan Li, Shichao Liu, Xiaoxiong Li, Kaige Cui, Lina Shang, Tianyou Cheng, Zhichao Zhang, Yingxiang Hou, Xiaozhu Yang, Hong Yan, Liqiang Duan, Zhou Tong, Changxin Wu, Zhida Liu, Shan Gao, Shu Zhuo, Weijin Huang, George Fu Gao, Jianxun Qi, Guijun Shang.

Corresponding authors:

huangweijin@nifdc.org.cn, gaof@im.ac.cn, jxqi@im.ac.cn, or guijun_shang@saari.org.cn.

This PDF file includes:

Figures S1 to S12

Tables S1

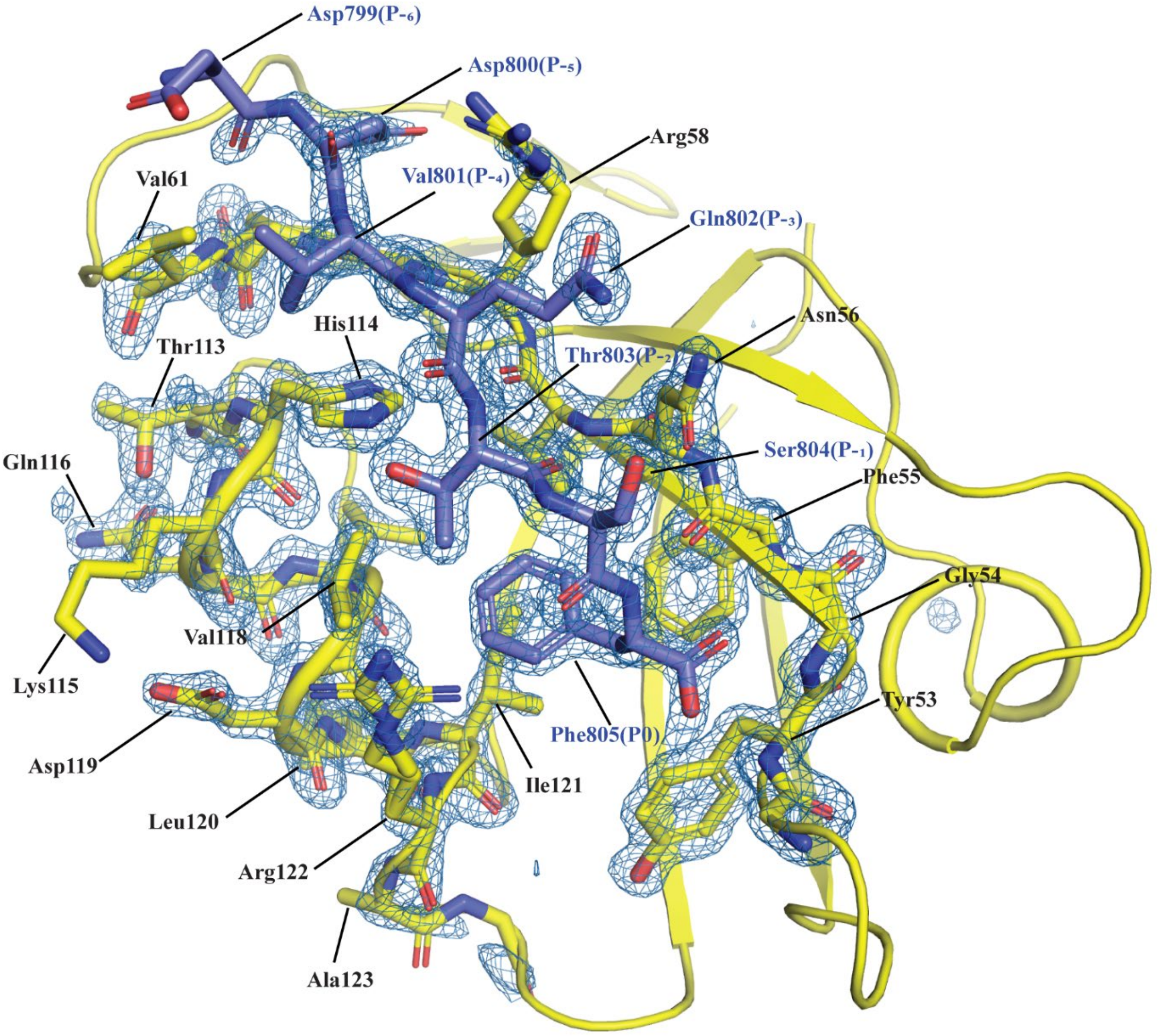

Fig. S1. 2Fo-Fc map of interface of ACE2-PBM/SNX27-PDZ complex.
Contour level is 2σ. The residues referred in the text are labeled.

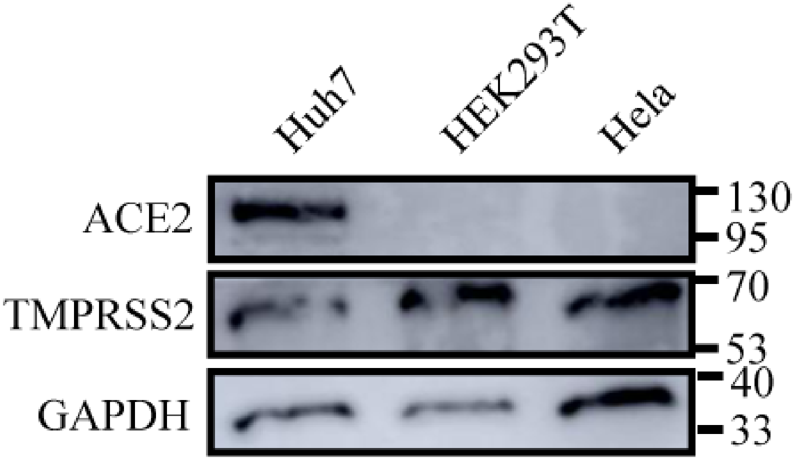

Fig. S2. The expression of ACE2 and TMPRSS2 in Huh7, HEK293T and Hela cells.
The cell samples were analyzed by immunoblotting with anti-ACE2, anti-TMPRSS2 and anti-GAPDH (loading control) antibodies.

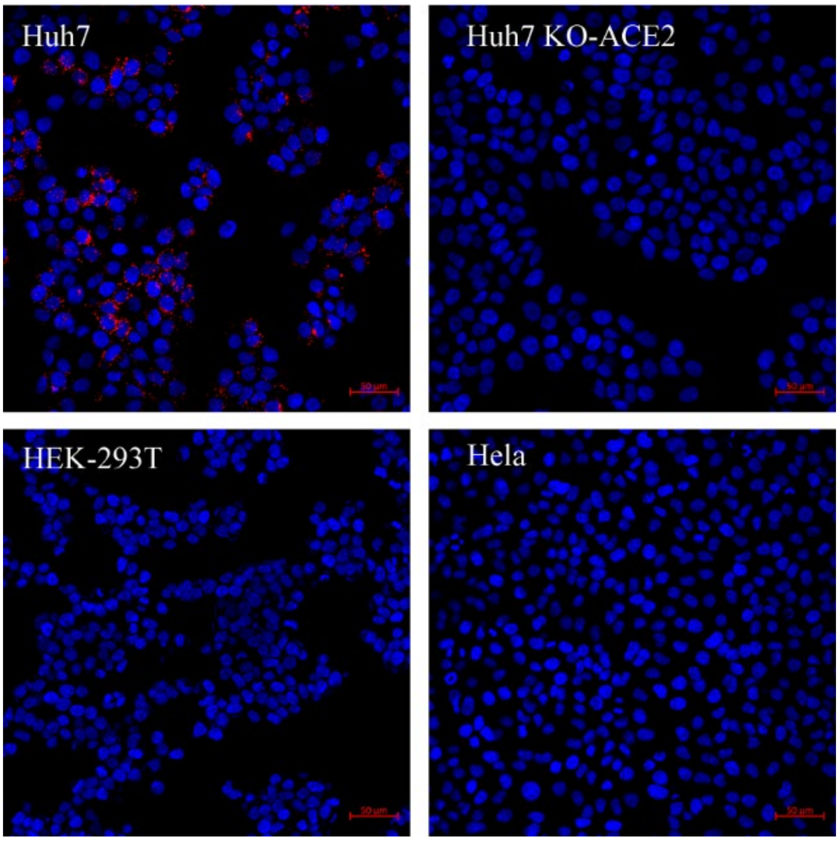

Fig. S3. The Specificity of RBD-AF555 protein.

Huh7 WT, Huh7 ACE2 KO, HEK-293T and Hela cells were treated with 1μM RBD-AF555 and incubated in the 37°C for 1 h. Then, the cells were fixed. Nuclei were counterstained with Hoechst 33342. Scale bars, 50 μm.

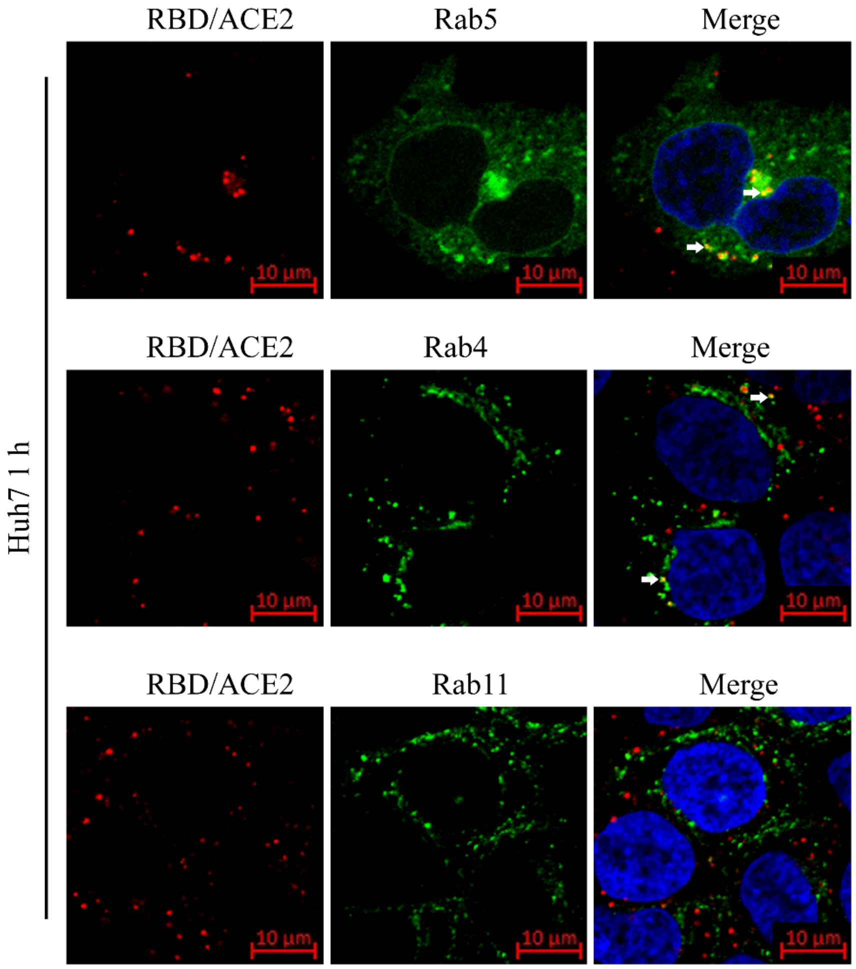

Fig. S4. Co-localization of RBD /ACE2 and Rab Protein in Huh7 cells.

GFP-Rab4, GFP-Rab5, GFP-Rab11 Huh7 cells were treated with 1μM RBD-AF555 and incubated at 37°C for 1 h. Then, the cells were fixed. Cell nuclei were counterstained with Hoechst 33342. Scale bars, 10 μm.

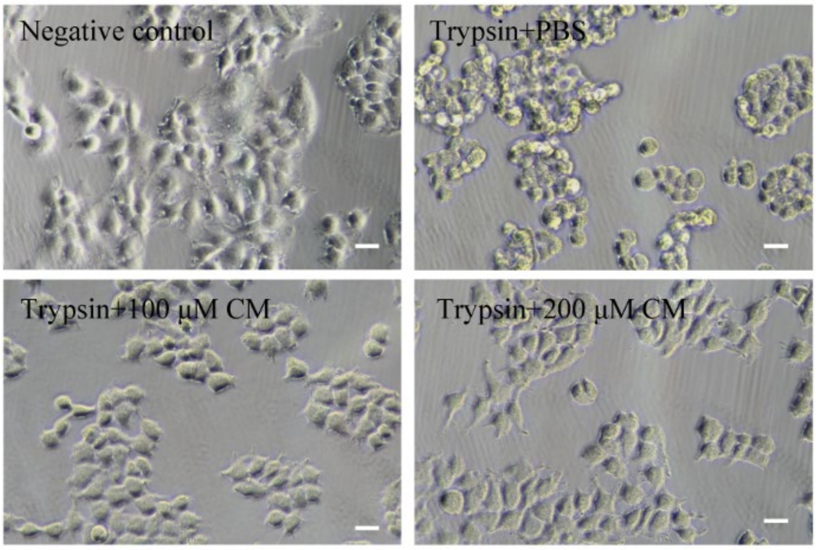

Fig. S5. Camostat mesylate (CM, a trypsin-like protease inhibitor) can inhibit the digestion of trypsin in Huh7 cells.

Huh7 cells were digested by trypsin without or with supplementation of Camostat mesylate (100μM and 200μM) and incubated at 37°C for 3 min (Magnification, ×200). Scale bars, 10 μm.

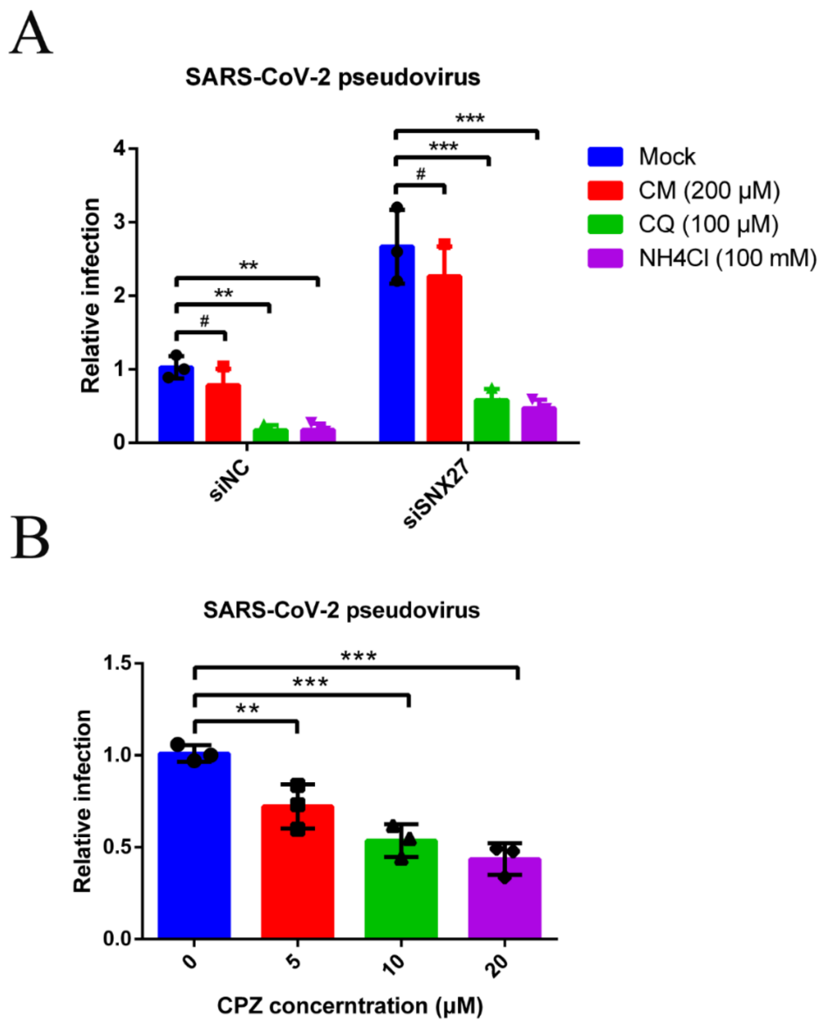

Fig. S6. Effects of lysosomotropic agents, SNX27 and clathrin-mediated endocytosis inhibitor on the infection of SARS-CoV-2 pseudovirus in Huh7 cells and infection was analyzed without surface ACE2 normalization.

(*A*) Huh7 siSNX27 and siNC cells were pretreated with 200 μM CM, 100μM CQ, and 100 mM NH_4Cl for 3 h before SARS-CoV-2 pseudovirus infection. After 24 h post-infection (hpi), the cells were lysed and the infectivities of the pseudoviruses are represented as luciferase activities. (*B*) Huh7 WT cells were pretreated with indicated concentrations of CPZ for 3 h before SARS-CoV-2 pseudovirus infection. After 24 h post-infection (hpi), the cells were lysed and the infectivities of the pseudoviruses are represented as luciferase activities. The data represent the mean ± SD of three independent experiments. $^{**}P < 0.01$, $^{***}P < 0.001$, $^{\#}P > 0.05$.

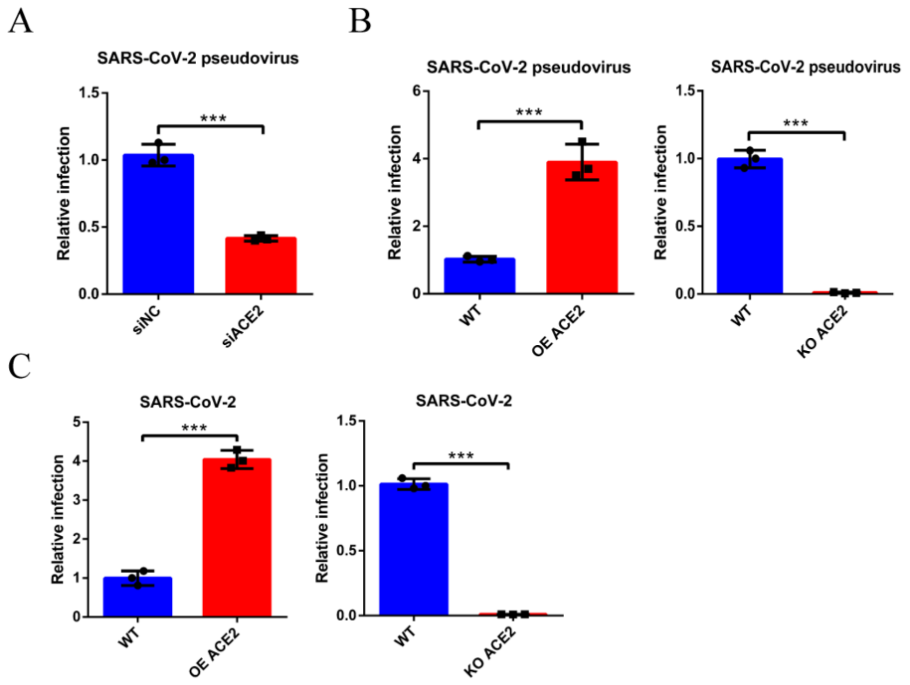

Fig. S7. The effect of the ACE2 disturbance on the infection of pseudo and authentic SARS-CoV-2.

(*A*) Huh7 siACE2 and siNC cells were infected with SARS-CoV pseudovirus for 24 h. The cells were lysed and the infectivities of the pseudoviruses are represented as luciferase activities. (*B*) Huh7 WT, ACE2 KO and ACE2 OE cells were infected with SARS-CoV-2 pseudovirus for 24 h. The cells were lysed and the infectivities of the pseudoviruses are represented as luciferase activities. (*C*) Huh7 WT, ACE2 KO and ACE2 OE cells were infected with SARS-CoV-2 at an MOI of 1. The relative concentration of viral RNA present in the supernatant at 16 hpi was determined by Real-time quantitative PCR analysis. The data represent the mean ± SD of three independent experiments. ***$P < 0.001$.

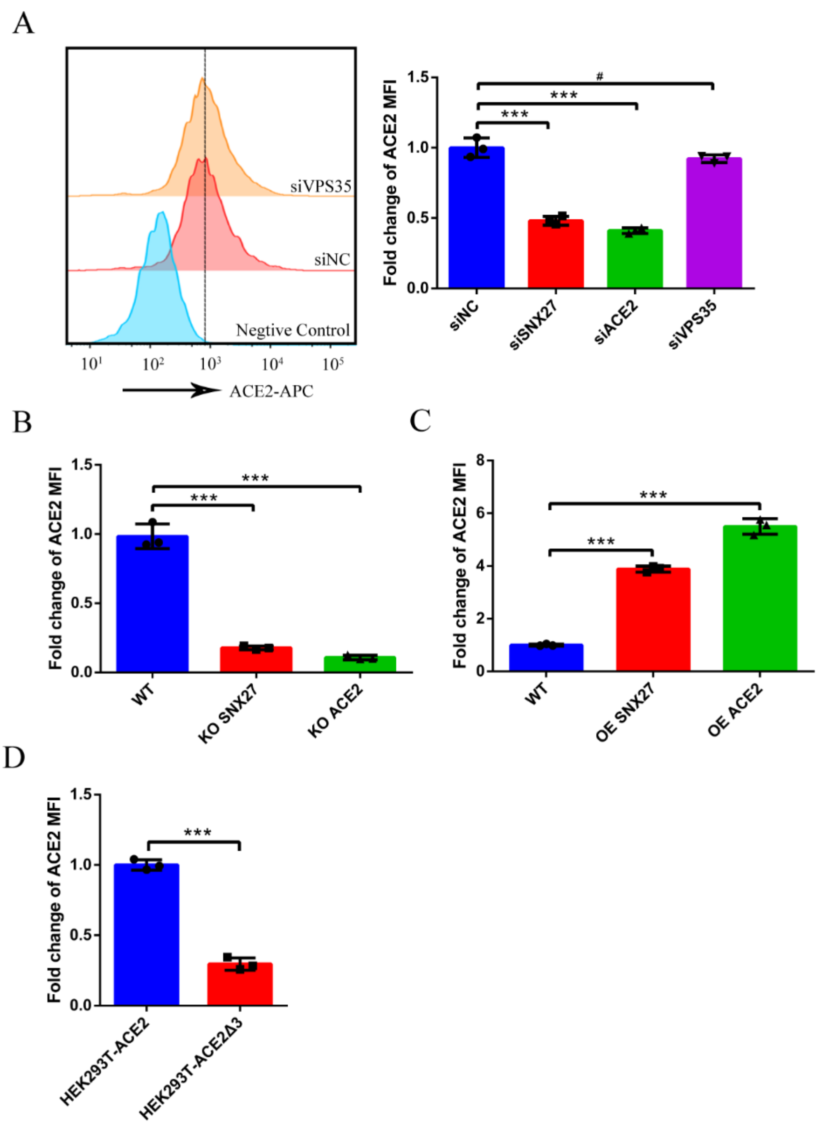

Fig. S8. Determination of cell surface expression of ACE2 by Mean Fluorescence Intensity (MFI) of flow cytometry analysis.

(*A*) Surface expression of receptor ACE2 was detected in siSNX27, siVPS35 and siACE2 Huh7 cells compared with siNC Huh7 cells. Representative histograms of flow cytometry analysis to determine cell surface expression of ACE2. Fold change of ACE2 surface MFI was shown and data are pooled from three independent experiments, quantified, and normalized to the controls of individual experiments. (*B*) Surface expression of receptor ACE2 was detected in SNX27 and ACE2 Knockout (KO) Huh7 cells compared with wild type (WT) Huh7 cells. Fold change of ACE2 surface MFI was shown and data are pooled from three independent experiments, quantified, and normalized to the controls of individual experiments. (*C*) Surface expression of receptor ACE2 was detected in SNX27 and ACE2 overexpression (OE) Huh7 cells compared with wild type (WT) Huh7 cells. Fold change of ACE2 surface MFI was shown and data are pooled from three independent experiments, quantified, and normalized to the controls of individual experiments. (*D*) Surface expression of receptor ACE2 was detected in HEK293T-ACE2 and HEK293T-ACE2Δ3 cells. Fold change of ACE2 surface MFI was shown and data are pooled from three independent experiments, quantified, and normalized to the controls of individual experiments. $^{***}P < 0.001$, $^{\#}P > 0.05$.

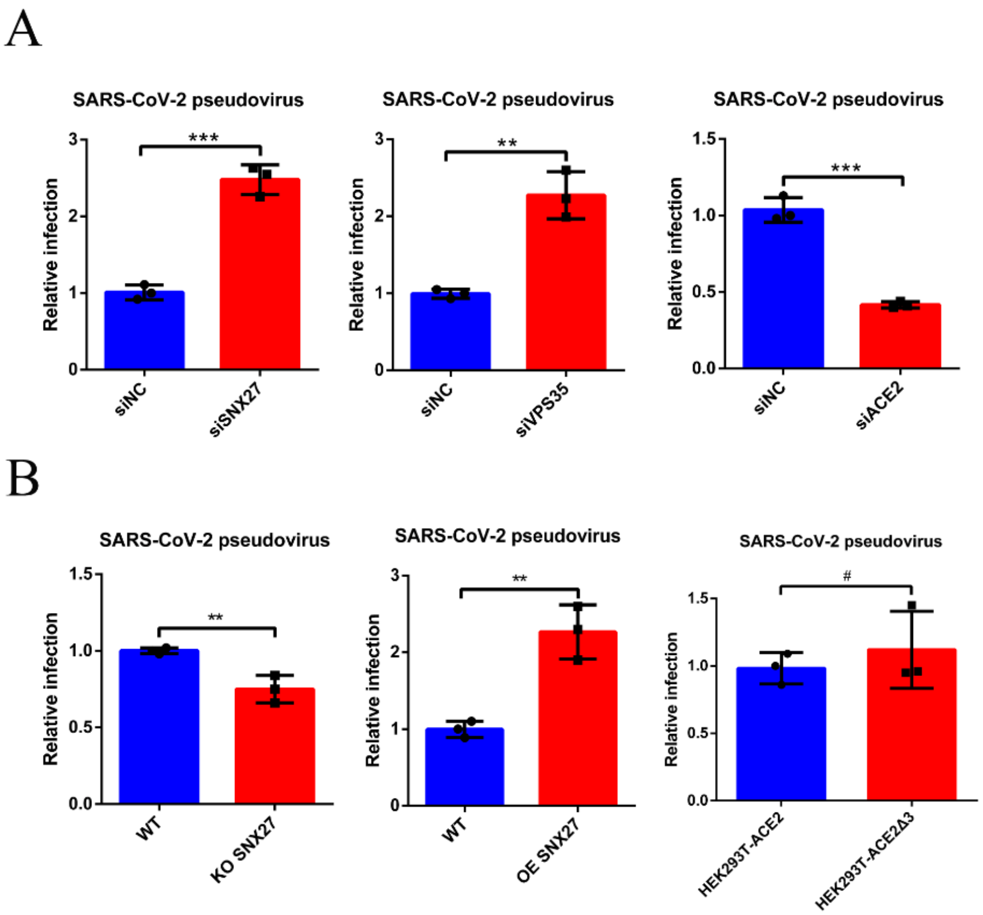

Fig. S9. The role of the SNX27 and VPS35 on the infection of pseudovirus of SARS-CoV-2 and infection was analyzed without surface ACE2 normalization.
(*A*) Huh7 siSNX27, siVPS35, siACE2 and siNC cells were infected with SARS-CoV-2 pseudovirus for 24 h. The cells were lysed and the infectivities of the pseudoviruses are represented as luciferase activities. (*B*) Huh7 WT, KO SNX27, OE SNX27 cells were infected with SARS-CoV-2 pseudovirus for 24 h. HEK293T-ACE2 and HEK293T-ACE2Δ3 cells were also infected with SARS-CoV-2 pseudovirus for 24 h. The cells were lysed and the infectivities of the pseudoviruses are represented as luciferase activities. The data represent the mean ± SD of three independent experiments. $^{**}P < 0.01$, $^{***}P < 0.001$, $^{\#}P > 0.05$.

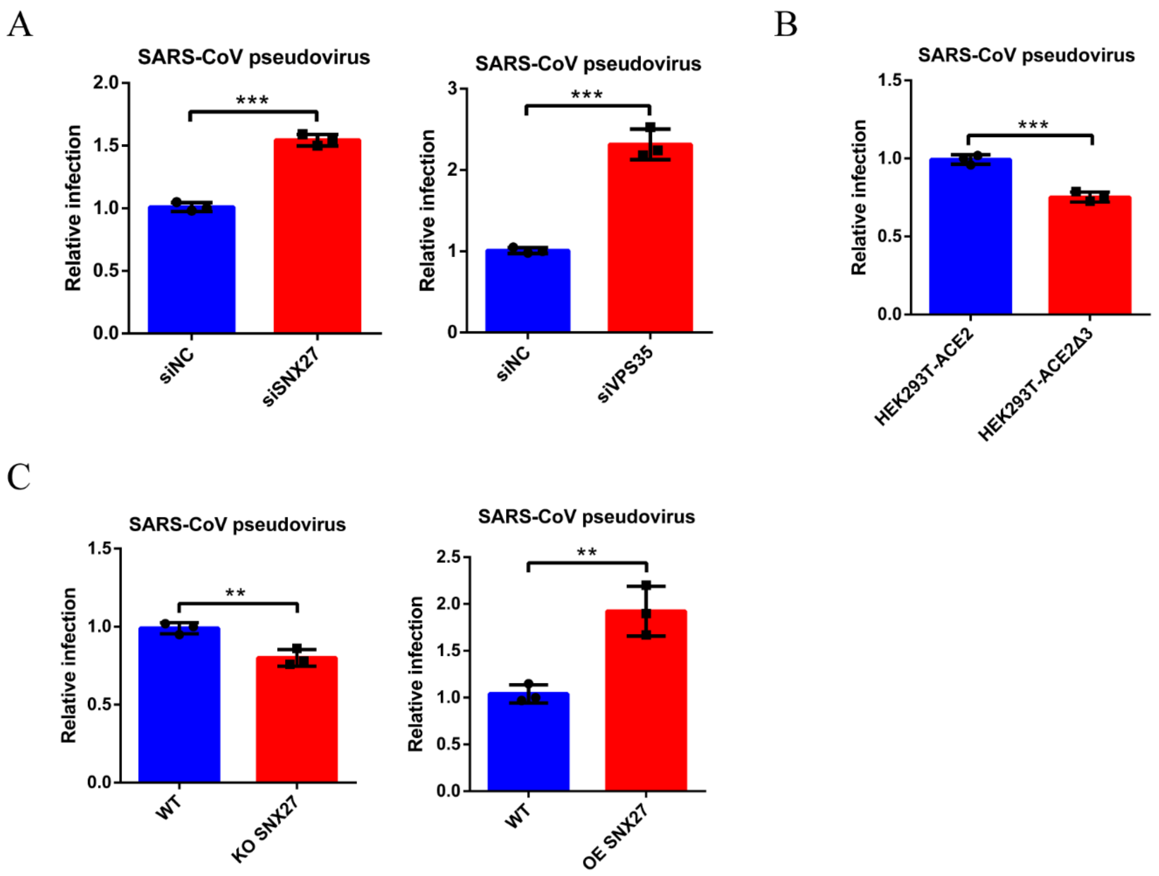

Fig. S10. The effect of the SNX27 and VPS35 on the infection of SARS-CoV pseudoviruses and infection was analyzed without surface ACE2 normalization.

(*A*) Huh7 siSNX27, siVPS35 and siNC cells were infected with SARS-CoV pseudovirus for 24 h. The cells were lysed and the infectivities of the pseudoviruses are represented as luciferase activities. (*B*) HEK293-ACE2 and HEK293-ACE2Δ3 cells were also infected with SARS-CoV pseudovirus for 24 h. (*C*) Huh7 WT, KO SNX27, OE SNX27 cells were infected with SARS-CoV pseudovirus for 24 h. The cells were lysed and the infectivities of the pseudoviruses are represented as luciferase activities. The data represent the mean ± SD of three independent experiments. $^{**}P < 0.01$, $^{***}P < 0.001$.

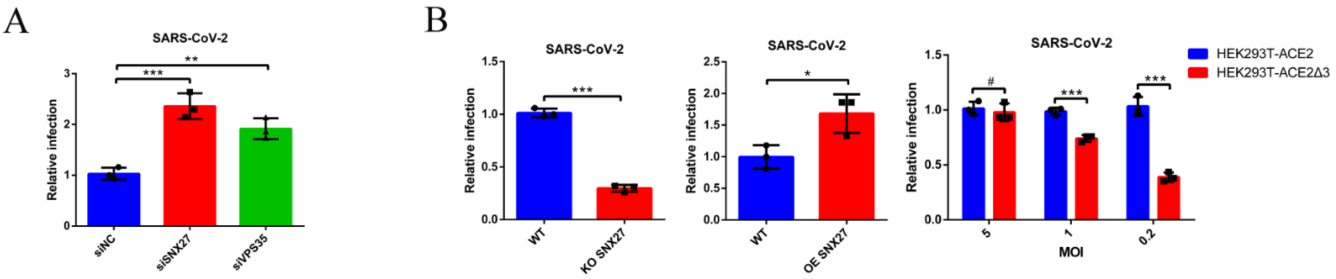

Fig. S11. The role of the SNX27 and VPS35 on the infection of authentic SARS-CoV-2 and infection was analyzed without surface ACE2 normalization.

(*A*) Huh7 siSNX27, siVPS35 and siNC cells were infected with SARS-CoV-2 at an MOI of 1. The relative concentration of viral RNA present in the supernatant at 16 hpi was determined by Real-time quantitative PCR analysis. (*B*) Huh7 WT, KO SNX27 and OE SNX27 cells were infected with SARS-CoV-2 at an MOI of 1. HEK293-ACE2 and HEK293-ACE2Δ3 cells were also infected with SARS-CoV-2 (MOI=5, 1, 0.2) for 16 h. The relative concentration of viral RNA present in the supernatant at 16 hpi was determined by Real-time quantitative PCR analysis. The data represent the mean ± SD of three independent experiments. $*P < 0.05$, $**P < 0.01$, $***P < 0.001$, $^{\#}P > 0.05$.

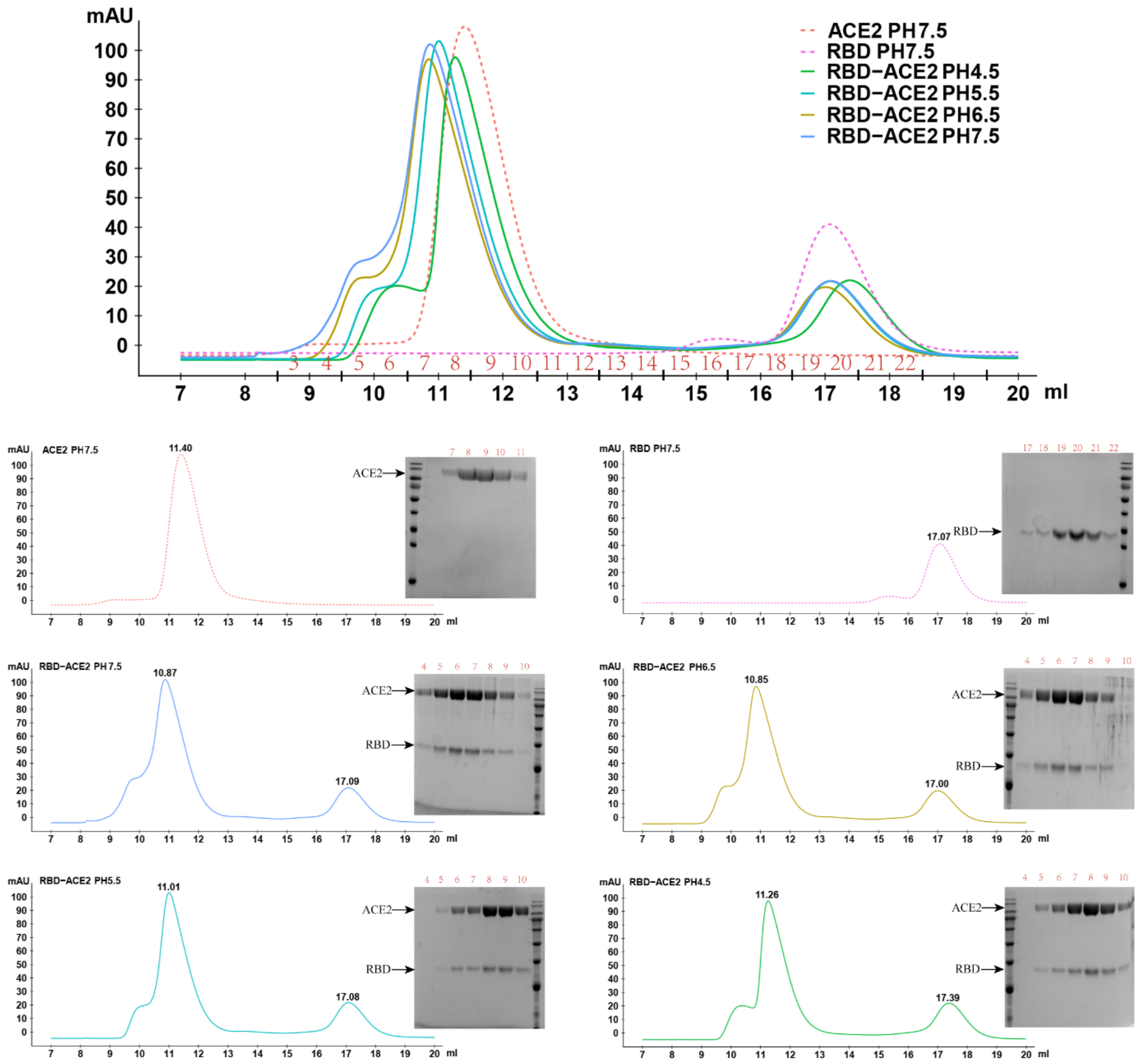

Fig. S12. The effect of pH on the stability of RBD/ACE2 complex.

The RBD (residues 331-531) / ACE2 (residues 17-741) complex is stable under acidic environments. RBD/ACE2 complex stability was examined using gel filtration chromatography on column superdex200 10X300 under different pH condition (pH 4.5, pH 5.5, pH 6.5 and pH 7.5). As for the gel filtration profile, the vertical axis shows the absorbance at 280 nm with arbitrary unit (mAU) and the horizontal axis stands for the elution volume. Co-migration of RBD and ACE was detected by SDS-PAGE and coomassie blue staining. The red number strands for the corresponding fraction numbers. ACE2 and RBD proteins were indicated by arrows.

Table S1. Data collection and refinement statistics

	ACE-PBM/SNX27-PDZ
**Data collection**	
Space group	H3
Cell dimensions	
a, *b*, *c* (Å)	88.55, 88.55, 43.27
α, β, γ (°)	90, 90, 120
Wavelength (Å)	0.979
Resolution (Å)	50-1.29 (1.32-1.29)
R_{merge}	0.055 (1.167)
$I / \sigma I$	17.3 (1.2)
CC1/2	0.998 (0.519)
Completeness (%)	97.8 (81.2)
Redundancy	8.6 (4.6)
**Refinement**	
Resolution (Å)	20.82-1.29
No. reflections	30746
R_{work} / R_{free}	0.1842/0.1906
No. atoms	
Protein	795
Ligand/ion	0
Water	134
B-factors	
Protein	29.9
Ligand/ion	
Water	37.8
R.m.s. deviations	
Bond lengths (Å)	0.003
Bond angles (°)	0.544
Ramachandran plot	
Favored (%)	97.98
Allowed (%)	2.02
Outliers (%)	0

*Values in parentheses are for highest-resolution shell.
